# Polydopamine-coated gold nanostar for combined antitumor and antiangiogenic therapy in multidrug-resistant breast cancer

**DOI:** 10.7150/ntno.36842

**Published:** 2019-06-06

**Authors:** You-Hong You, Yu-Feng Lin, Bhanu Nirosha, Huan-Tsung Chang, Yu-Fen Huang

**Affiliations:** 1 Department of Biomedical Engineering and Environmental Sciences, National Tsing Hua University, Hsinchu 30013, Taiwan, ROC; 2 Department of Chemistry, National Taiwan University, Taipei 10617, Taiwan, ROC

**Keywords:** Gold nanostar, Polydopamine, Antiangiogenesis, Cancer combination therapy, Drug delivery, Multidrug resistance

## Abstract

Cancer combination therapy can improve treatment efficacy and is widely utilized in the biomedical field. In this paper, we propose a facile strategy to develop a polydopamine (PDA)-coated Au nanostar (NS@PPFA) as a multifunctional nanoplatform for cancer targeting and combination therapy. The Au nanostar demonstrated high photothermal conversion efficiency because of the tip-enhanced plasmonic effect. Modification of PDA and folic acid on the NS surface improved its drug-loading efficiency and targeting capability. In vitro, compared with nontargeted cells, targeted breast cancer MCF-7 cells demonstrated efficient uptake of chemodrug-loaded NS-D@PPFA through the receptor-mediated endocytosis pathway. In combination with the photothermal effect induced by near-infrared laser irradiation, controlled payload release could be activated in response to both internal (acid) and external (photothermal) stimuli, leading to an efficient chemo-photothermal action against MCF-7 cells and drug-resistant MCF-7/ADR cells. By contrast, cellular damage was less obvious in normal HaCaT (human skin keratinocytes) and NIH-3T3 cells (murine fibroblasts). In addition, payload-free NS@PPFA exhibited a high binding affinity (*K*_d_ = 2.68 × 10^-10^ M) toward vascular endothelial growth factor (VEGF-A165), which was at least two orders of magnitude stronger than that of highly abundant plasma proteins, such as human serum albumin. Furthermore, in vitro study showed that NS@PPFA could effectively inhibit VEGF-A165-induced proliferation, migration, and tube formation of human umbilical vein endothelial cells, resulting in additional therapeutic benefits for eradicating tumors through a simultaneous antiangiogenic action in chemo-photothermal treatment. The combined treatment also exhibited the lowest microvessel density, leading to a potent antitumor effect in vivo. Overall, our “all-in-one” nanoplatform is highly promising for tumor therapy, enabling effective treatment against multidrug-resistant cancers.

## Introduction

Photothermal therapy (PTT) is a promising approach for treating cancer because of its minimally invasive nature and few side effects.^1^, ^2^ Nanoagents showing high absorption in the near-infrared (NIR) region and excellent photothermal conversion efficiencies can be used for PTT.^3^, ^4^ Gold nanorod^5^, ^6^ and gold nanostar (Au NS),^7^, ^8^ which can efficiently convert light energy into heat, are representative candidates that can be used for the thermal ablation of tumors. However, PTT still has some limitations. For example, heat produced by the incident beam decreases from the center, and adjacent cells damaged by mild hyperthermia might be repaired by heat shock proteins, resulting in recurrence of the residual tumor.^9^ To overcome these challenges, combination therapy that possesses the collective merits of respective individual treatments has attracted considerable attention recently. Chemo-PTT is one of the typical approaches used in combination therapy.^10^-^12^ The immediate damage caused by the hyperthermic environment and the long-lasting chemotherapy effect exerted by the release of drug payload can achieve the synergistic effect, increasing the efficiency of cancer treatment.

Although the therapeutic efficacy of chemo-PTT is more satisfactory than that of a single treatment alone, poor penetration and heterogeneous distribution of drugs in a tumor are other major obstacles that can cause an inevitable depth-dependent decline of treatment efficacy.^13^ Furthermore, in a multidrug-resistant (MDR) tumor, the efflux pumping of drugs by P-glycoprotein may compromise the therapeutic efficacy of a chemo-combined therapy,^14^ suggesting the need of a more effective approach to overcome resistance. Antiangiogenic therapy that mainly attempts to perturb the angiogenic potential of tumor vascular endothelial cells (ECs) is a promising strategy.^15^, ^16^ Blocking the binding of growth factors to their receptors leads to a significant decrease in neovascularization. This can efficiently inhibit tumor growth because it results in the shortage of nutrients and oxygen supplied by vessel capillaries. Moreover, ECs can be more easily accessed compared with tumor cells that are distant to tumor vessels. Combining the treatment of tumors and tumor vascular ECs might be more advantageous for promoted drug uptake and improved treatment efficacy in drug-resistant cancers.^17^

Nanomaterials, such as gold nanoparticle (Au NP) and graphene oxide (GO), have been reported as potential antiangiogenic agents because they interfere with the signaling pathway involved in angiogenesis.^18^, ^19^ For example, Mukherjee et al. reported that Au NP exert an antiproliferative effect on ECs by direct binding to vascular endothelial growth factor (VEGF) through the sulfur/amines present in the amino acids of the heparin-binding domain (HBD).^18^ Bovine serum albumin-capped GO also effectively disturb the angiogenesis process in vivo via an ultrastrong VEGF adsorption and its activity suppression.^19^ These findings suggests that an efficacious binder may initiate a conformational change mediated by a direct VEGF contact, leading to an effective inhibition of subsequent triggering of angiogenesis.^19^, ^20^ Moreover, nanocarriers possessing dual anticancer and antiangiogenic activities have been developed to achieve higher efficiency of cancer treatment.^21^-^23^ Li et al. constructed RGD-conjugated mesoporous silica nanoparticles for doxorubicin (DOX) and combretastatin A4 loading.^24^ While being specifically bound to tumor cells and vasculatures, drug payloads can be sequentially released in an acidic tumor environment. Such a dual-modality nanoagent can eventually lead to the synergistic inhibition of tumor proliferation and vascular formation.

In this study, we developed a multifunctional nanocarrier based on polydopamine (PDA)-grafted Au NS. Au NS contain multiple sharp branches, which can enhance the local electromagnetic field,^25^ yielding a higher NIR photothermal conversion efficiency than other Au NP shapes. Surfactant-free synthetic routes have also been developed for Au NS to greatly improve their biocompatibility and facile functionalization 25-26. On the other hand, the mussel-inspired PDA coating^27^-^30^ not only enables effective drug loading through electrostatic or π-π stacking interactions but also provides robust photothermal stability to spiky Au NS, significantly improving their photothermal efficiency.^31^ After modification with a targeting ligand, namely folic acid-tethered thiol polyethylene glycol (HS-PEG-FA), the resulting nanocomposite NS-D@PPFA causes dual chemo-photothermal injuries on targeted MCF-7 breast cancer cells and drug-resistant ADR sublines in response to NIR irradiation. Furthermore, NS-D@PPFA could inhibit not only the growth of tumor cells but also the proliferation of tumor vascular ECs. The mechanism of action refers to the effective trapping of the growth factor VEGF by an electrostatic interaction, possibly between the negatively charged PDA and the basic heparin-binding domain (HBD) of VEGF. Therefore, a blockade of in vivo VEGF-mediated angiogenesis can significantly reduce vascular density. When given in combination with PTT and chemotherapy, the anti-VEGF function of PDA-NS found in this study may represent an effective treatment approach for MDR tumors. To the best of our knowledge, this is the first study to reveal an active role of PDA-NS in antiangiogenic cancer therapy.

## Results and Discussions

**Scheme 1:**
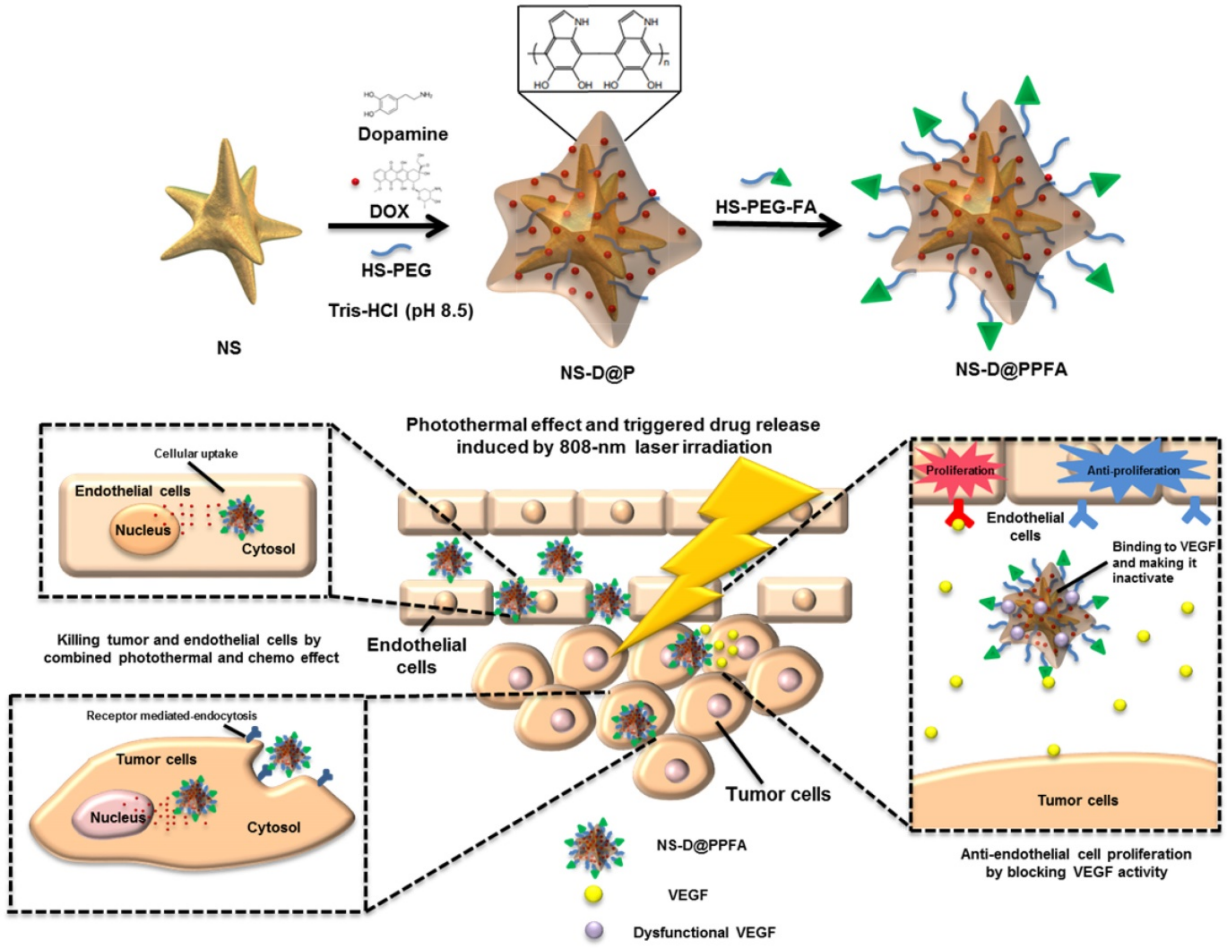
Schematic illustration of the preparation procedure and the combined antitumor and antiangiogenic mechanisms of NS-D@PPFA.

**Synthesis of NS-D@PPFA.** The design and synthetic strategy of NS-D@PPFA are illustrated in **Scheme 1**. The photothermal nanoconverter Au NS was synthesized using HEPES as a reducing and growth-directing agent.^26^ The concentration of the as-prepared NS was determined to be 1.6 nM and denoted as 4×. To achieve an effective DOX loading and controlled drug release, PDA was grafted on the surface of NS through the self-polymerization reaction of dopamine in an alkaline condition. The simultaneous incorporation of HS-PEG also stabilized NS during the polymerization step. Moreover, to improve the specificity toward and uptake efficiency of targeted cancer cells, HS-PEG-FA was conjugated to the surface of PDA through the Michael addition reaction.^32^ The functionalization of HS-PEG-FA resulted in prolonged blood circulation time through steric repulsion and the stealth effect.^33^ Afterwards, NS-D@PPFA could extravasate to the tumor site through the enhanced permeability and retention (EPR) effect and bind to folate receptors overexpressed by cancer cells for subsequent internalization. Under NIR laser irradiation, the dual pH and temperature responsiveness of NS-D@PPFA enabled controlled release of equipped drug payloads, leading to a simultaneous chemo-photothermal action to eradicate tumor cells. Concurrently, the anti-VEGF effect of NS-D@PPFA was exerted on tumor vascular ECs to reduce tumor angiogenesis. PDA-NS-enabled antitumor and antiangiogenic combination therapy may help in the development of innovative strategies for treating MDR cancer.

**Figure 1:**
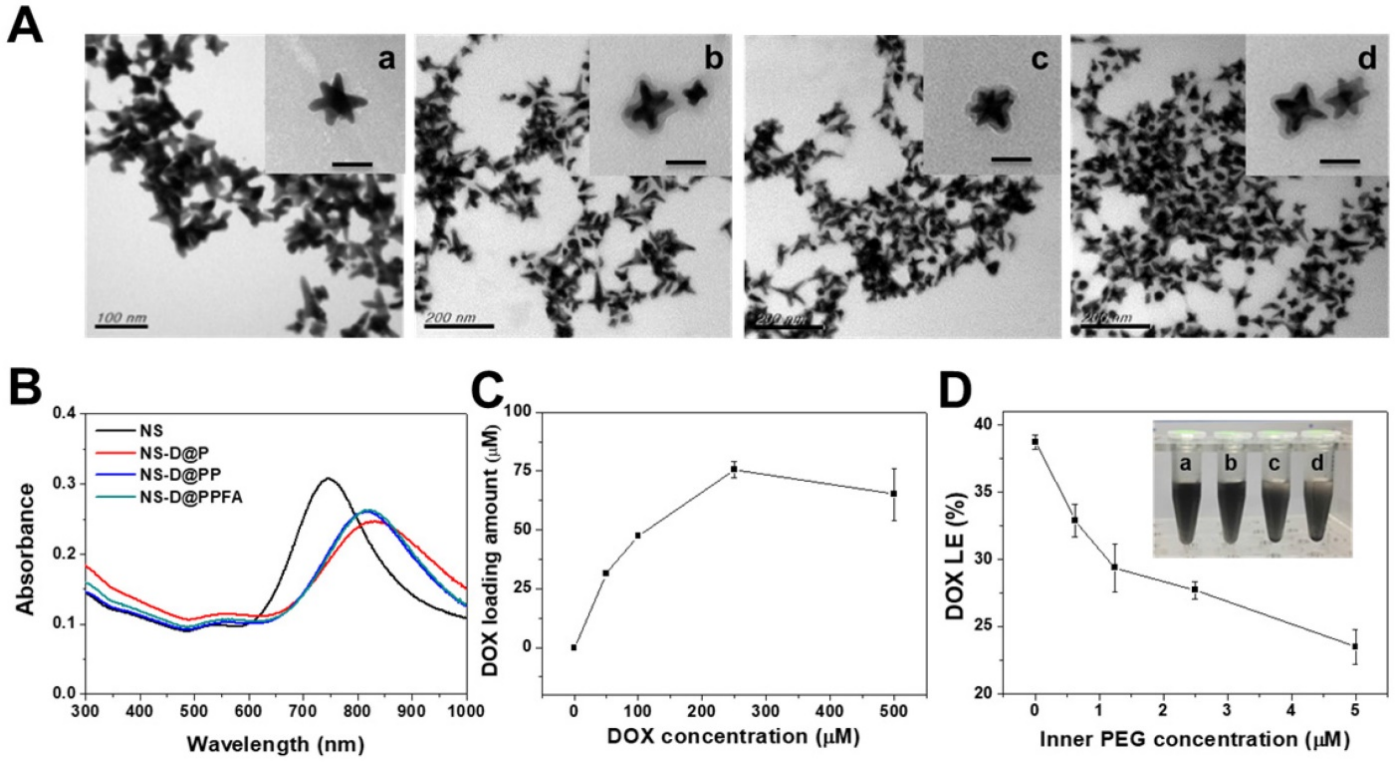
Characterization of various Au NS-based therapeutic nanoagents. (A) TEM images of (a) NS, (b) NS-D@P, (c) NS-D@PP, and (d) NS-D@PPFA, respectively. (Scale bar: 50 nm for enlarged photograph). (B) The UV-Vis spectra of different nanocomposites. (C) The loading amount of DOX on NS-D@PPFA in the presence of various concentration of DOX (0 - 500 μM). (D) The DOX LE (%) of NS-D@PPFA under various concentration of inner PEG. Photograph showed the dispensability of NS-D@PPFA suspension with (a) 5, (b) 2.5, (c) 1.25 and (d) 0.625 μM inner PEG during the polymerization step. The NS concentration in each test is fixed at 1.6 nM.

**Characterization of NS-D@PPFA.** As shown in **Table 1**, NS exhibited a hydrodynamic size of 78 ± 3.4 nm, containing a branched-like structure visible through transmission electron microscopy (TEM; image a in **Figure 1A**). The negative surface zeta potential (-28 ± 1.8 mV, **Table 1**) was ascribed to the coating of HEPES,^34^ which stabilized NS in aqueous solution. In the UV-Vis spectrum (**Figure 1B**), NS demonstrated strong absorption at a wavelength of 750 nm. This NIR absorption peak was attributed to the surface plasmon resonance (SPR) of Au NS tips.^26^ After surface modification by the grafting of PDA, a uniform assembled layer with a thickness of approximately 10 nm was observed through TEM (images b-d in **Figure 1A**). The SPR peak of NS-D@P exhibited a red-shifted and broadening profile (**Figure 1B**) compared with that of bare NS, indicating the corresponding change of the local refractive index after PDA coating.^35^ The subsequent modification of HS-PEG-FA resulted in an SPR peak at a similar wavelength but with less peak broadening, suggesting an improvement in colloidal dispersibility. Moreover, according to the binding isotherm (**Figure S1A**), the maximum amount of PEGylated FA attached to each nanoparticle was approximately 6000. Therefore, NS-D@PPFA could be more efficiently dispersed in phosphate-buffered saline (PBS) compared with NS-D@P (**Figure S1B**), and no significant change in hydrodynamic size was observed in the culture medium (DMEM supplemented with 10% FBS) after incubation for 48 h (**Figure S1C**). As summarized in **Table 1**, all PEGylated nanocomposites had a hydrodynamic size of approximately 150 nm and a zeta potential of approximately -20 mV. By contrast, non-PEGylated NS-D@P had a hydrodynamic size of 233 ± 25 nm and a zeta potential of -3 ± 0.6 mV. This result again confirmed that successful PEG grafting can enhance colloidal stability and that a resultant hydrodynamic size of <200 nm can lead to efficient tumor accumulation and cellular uptake.^36^ In addition, a significant difference in physicochemical properties was not observed between NS-D@PPFA and NS-D@PP, indicating the applicability of using NS-D@PP as a nontargeted nanoagent in the following study.

As shown in **Figure 1C**, the drug-loading capacity of NS-D@PPFA was determined using a serial concentration of DOX, and the maximum loading amount reached approximately 75 μM for an NS of 1.6 nM. Compared with NS@PPFA, an increase in absorption ranging from 480 to 600 nm in the UV-Vis spectrum (**Figure S2**) supported successful DOX loading. No significant change in the hydrodynamic size and zeta potential was observed between NS@PPFA and NS-D@PPFA (**Table 1**), suggesting that the simultaneous drug encapsulation was attainable during the one-step synthesis of the PDA shell coating. In addition, the optimal DA concentration chosen for the following experiments was 0.25 mg/mL, which resulted in the highest red shift in the SPR peak and the largest hydrodynamic size owing to the thickest coating shell^31^ (**Figure S3**). With a further increase in the DA concentration (1.25 mg/mL), the resulting nanoparticles were found to exhibit a strong tendency toward self-aggregation.^37^ The DOX loading content (LC%) and loading efficiency (LE%) of NS-D@P (11.0% and 31.6%, respectively; **Table 1**) were higher than those of NS-D (1.0% and 1.8%, respectively), further suggesting that the diversified functional groups of PDA^38^ made it suitable for drug loading through π-π stacking, electrostatic interaction, and hydrogen binding.^29^ In addition, the detection of ignorable DOX leakage (<3%), followed by PEGylation, demonstrated its strong interaction with drug payloads. The coexistence of HS-PEG (represented as inner PEG) during the PDA grafting procedure could prevent undesirable NS aggregation (inset in **Figure 1D**). However, an increased level of inner PEG reduced the DOX LE (%) because of the occupancy of available binding sites (**Figure 1D**). Therefore, using the lowest amount of inner PEG (2.5 μM) that provided adequate steric repulsion to maintain the stability of NS was identified as the optimal operation condition.

**Figure 2:**
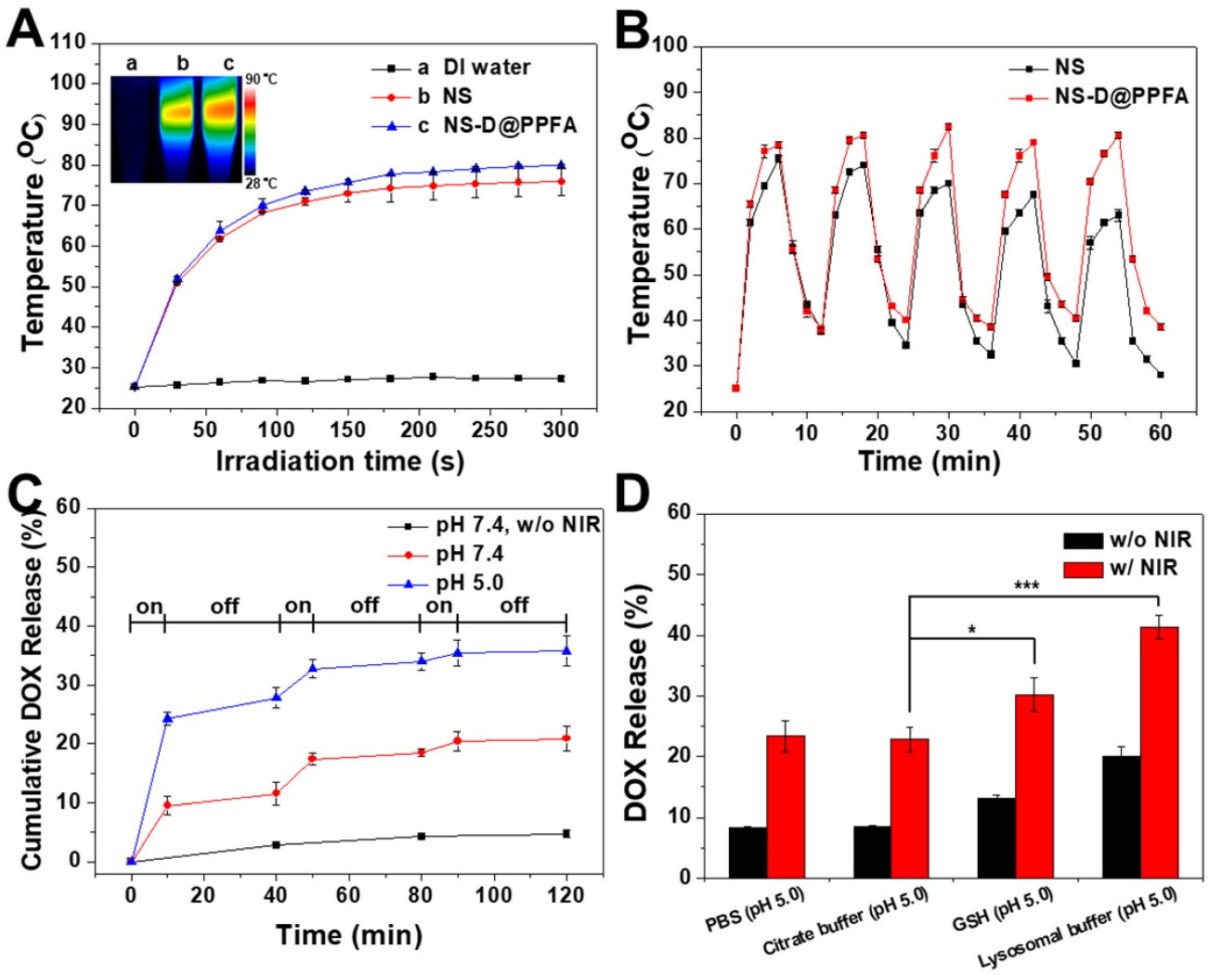
(A) Temperature curves versus time during irradiation with 808-nm laser (0.9 W/cm^2^) for Eppendorf tubes containing water, NS, and NS-D@PPFA (1.6 nM), respectively. Inset: Thermal camera images of each sample after 6 min irradiation. (B) The photothermal stability of NS and NS-D@PPFA (1.6 nM) under repeated laser irradiation (0.9 W/cm^2^). The samples were irradiated repeatedly over a period of 6 min, followed by 6 min intervals with the laser turned off. (C) Cumulative DOX release from NS-D@PPFA (1.6 nM) in PBS (pH 5.0 and 7.4, respectively) triggered by repeated NIR irradiation (0.9 W/cm^2^, 10 min). (D) DOX release from NS-D@PPFA (1.6 nM) in PBS (pH 5.0), citrate buffer (140 mM Na_2_HPO_4_, 60 mM citrate acid, pH 5.0), GSH (10 mM in citrate buffer, pH 5.0) and lysosomal buffer (23.5 mg/mL L-cysteine in citrate buffer, pH 5.0), respectively. Each sample was irradiated with NIR laser (0.9 W/cm^2^) for 10 min. *p < 0.05, ***p < 0.001.

**Photothermal effect and controlled drug release of NS-D@PPFA.** As shown in **Figure 2A**, the photothermal conversion ability of the developed nanoagent was evaluated. Under NIR laser irradiation (808 nm, 0.9 W/cm^2^), the temperature of NS and NS-D@PPFA increased rapidly to 75 and 78 °C, respectively, within 3 min, whereas no change occurred in the aqueous solvent. The visualization of a similar photothermal heating property was attributed to the equivalent optical density at 808 nm observed for two nanoparticle suspensions (**Figure 1B**). NS-D@PPFA also revealed a concentration-dependent temperature increase at the same irradiation dose (**Figure S4**). Moreover, the temperature profile of NS-D@PPFA remained unchanged under repeated irradiation cycles, whereas that of NS decayed gradually (**Figure 2B**). The absorption spectrum of NS-D@PPFA (**Figure S5)** was also retained considerably compared with the blue shift and the decrease in the SPR peak observed for bare Au NS after laser irradiation. Overall, these results demonstrate that PDA coating can prevent the deformation of nanospikes on Au NS to sustain its NIR responsiveness for an enhanced photothermal efficacy.^31^

We next examined the photothermally triggered DOX release from NS-D@PPFA in PBS at different pH values. As shown in **Figure 2C**, after 2 h of on-off laser irradiation, accumulated release of DOX in NS-D@PPFA reached 35% and 20% in PBS at pH 5.0 and 7.4, respectively. By contrast, drug leakage was negligible (<5%) in the dark during the detection period (2 h). The observation of a more efficient DOX release at a lower pH was attributed to the enhancement of electrostatic repulsion by the simultaneous protonation of both PDA and drug molecules under the acidic condition.^39^ In addition, when exposed to NIR irradiation, on-demand drug release occurred remotely through photothermal disruption of intermolecular forces,^40^ such as the hydrogen bond between NS-D@PPFA and DOX. PDA is a biodegradable polymer^41^, ^42^ that has a high propensity to interact with specific biomolecules or enzymes. Thus, we examined the release behavior of DOX from NS-D@PPFA in the lysosomal buffer (23.5 mg/mL L-cysteine in 140 mM Na_2_HPO_4_, 60 mM citrate acid, pH 5.0). L-cysteine was used to tune the redox potential (-220 mV) and mimic the lysosome environment.^43^ As shown in **Figure 2D,** the amount of DOX released in the lysosomal buffer was approximately two times higher than that released in PBS (pH 5.0) or citrate buffer (140 mM Na_2_HPO_4_, 60 mM citrate acid, pH 5.0) at the same pH value. This finding can be attributed to the existence of L-cysteine. Furthermore, the observation of a similar trend in 10 mM GSH (citrate buffer, pH 5.0) indicated the susceptibility of NS-D@PPFA toward thiol degradation,^44^, ^45^ enabling a more effective drug release in response to high levels of intracellular thiols. The UV-Vis spectrum in **Figure S6A** shows that when NS-D@PPFA (in the lysosomal buffer) was exposed to NIR laser irradiation, an obvious decrease and a blue shift in the SPR peak were observed. TEM images also revealed the shedding of PDA from the NS surface (inset in **Figure S6A**). Furthermore, a new peak appearing in the UV region was detected from the supernatant of the exposed sample (**Figure S6B**), indicating that the thiol degradation of PDA can be facilitated through photothermal heating.^31^ Collectively, the multiple-stimuli responsive design makes our nanoagent an ideal platform for spatiotemporally controlled photothermal combined chemotherapy.

**Figure 3:**
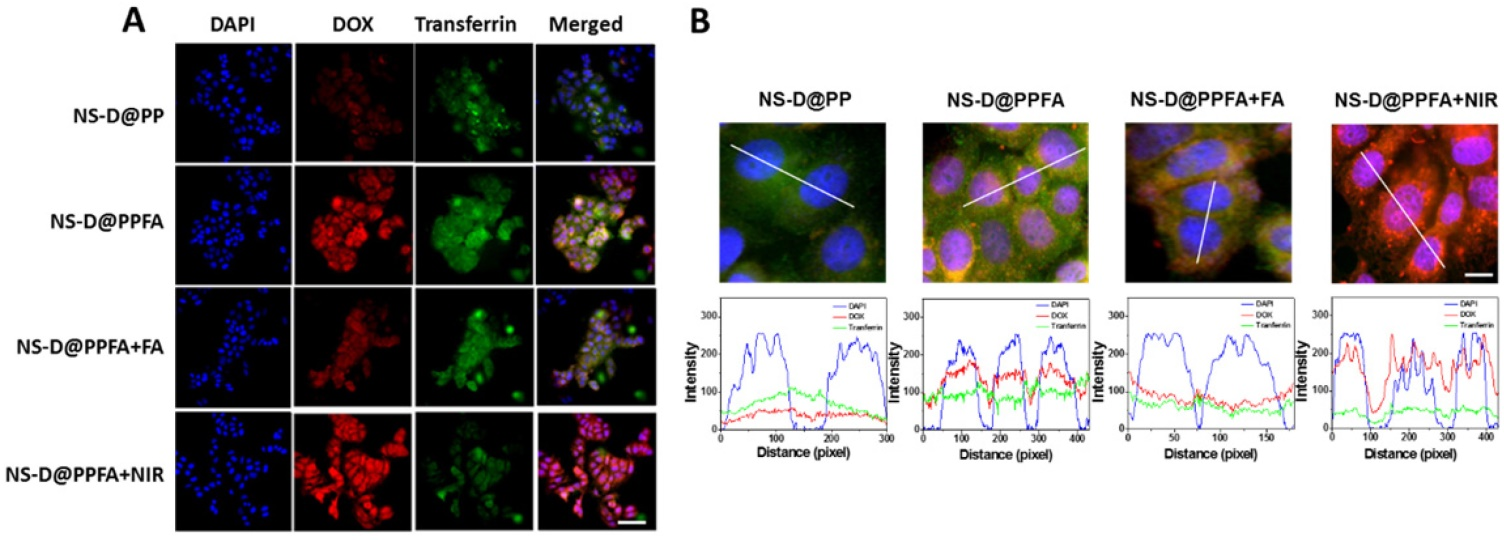
(A) Fluorescence and (B) confocal microscopic images after 2 h-cellular uptake of NS-D@PP, NS-D@PPFA, NS-D@PPFA+FA and NS-D@PPFA+NIR (0.8 nM) in MCF-7 cells. (Scale bar: 50 and 10 μm, respectively). Pixel intensities of DAPI (blue), DOX (red) and Alexa Fluor 633 conjugate transferrin (green) along a line were demonstrated by line-scan graphs. For competitive assay, cells were pre-treated with free FA (1 mM) for 1 h, followed by a subsequent incubation with NS-D@PPFA. For NIR exposure, cells treated with NS-D@PPFA were washed with fresh medium and irradiated with NIR laser (808 nm, 0.9 W/cm^2^) for 15 min.

**Cellular uptake and triggered drug release of NS-D@PPFA in vitro.** As shown in **Figure 3A**, the cellular uptake capabilities of NS-D@PP and NS-D@PPFA were investigated against folate receptor-overexpressing MCF-7 breast cancer cells^46^ through fluorescence microscopy. NS-D@PPFA exhibited a higher DOX fluorescence intensity than did NS-D@PP, indicating that the incorporation of FA in NS-D@PPFA can improve specific binding and uptake efficiency. Furthermore, to elucidate the mechanism underlying the interaction, a competitive binding assay was performed using a 125-fold molar excess of free FA. The fluorescence intensity of DOX significantly decreased within NS-D@PPFA-treated cells coincubated with FA, suggesting that the endocytosis pathway is mediated by the folate receptor.^32^ Similar results were obtained in flow cytometry (**Figure S7**); the relative fluorescence intensities of NS-D@PPFA and NS-D@PPFA plus FA were 145% and 119% versus NS-D@PP, respectively. In addition, as shown in **Figure S8**, the highest intracellular amounts of NS-D@PP, NS-D@PPFA, and NS-D@PPFA plus FA were 2.2, 22.4, and 2.1 pg Au/cell, respectively. Thus, collectively, NS-D@PPFA has the ability to bind to folate receptors on the surface of cancer cells, resulting in effective cellular internalization.

Subsequently, the intracellular distribution of DOX was investigated through confocal fluorescence microscopy. The fluorescent transferrin (green) and DAPI (blue) were employed to mark acidic organelles, such as endolysosomes, and nuclei, respectively. As shown in **Figure 3B**, NS-D@PP-treated cells exhibited a weaker DOX fluorescence intensity, whereas NS-D@PPFA-treated cells showed a stronger and higher degree of colocalization between DOX and DAPI under the same incubation condition. In addition to enhanced cellular uptake, this finding suggests that the binding to the folate receptor is effective for subsequent endolysosomal trafficking, leading to an acid-triggered release for nuclear drug accumulation. By contrast, the fluorescence intensity of DOX in cells exposed to NS-D@PPFA further increased after laser irradiation (**Figure 3 and S7**). A quantitative analysis of corresponding images exhibited a pronounced DOX signal (p = 0.02) but a decreased transferrin intensity (p = 0.02) (**Figure S9**), indicating that a more efficient endolysosomal escape and payload release are activated photothermally upon light exposure.^47^ Overall, NS-D@PPFA exhibits a dual responsiveness toward pH and NIR irradiation, leading to successful nucleus-targeted delivery of its drug cargos.

**Figure 4:**
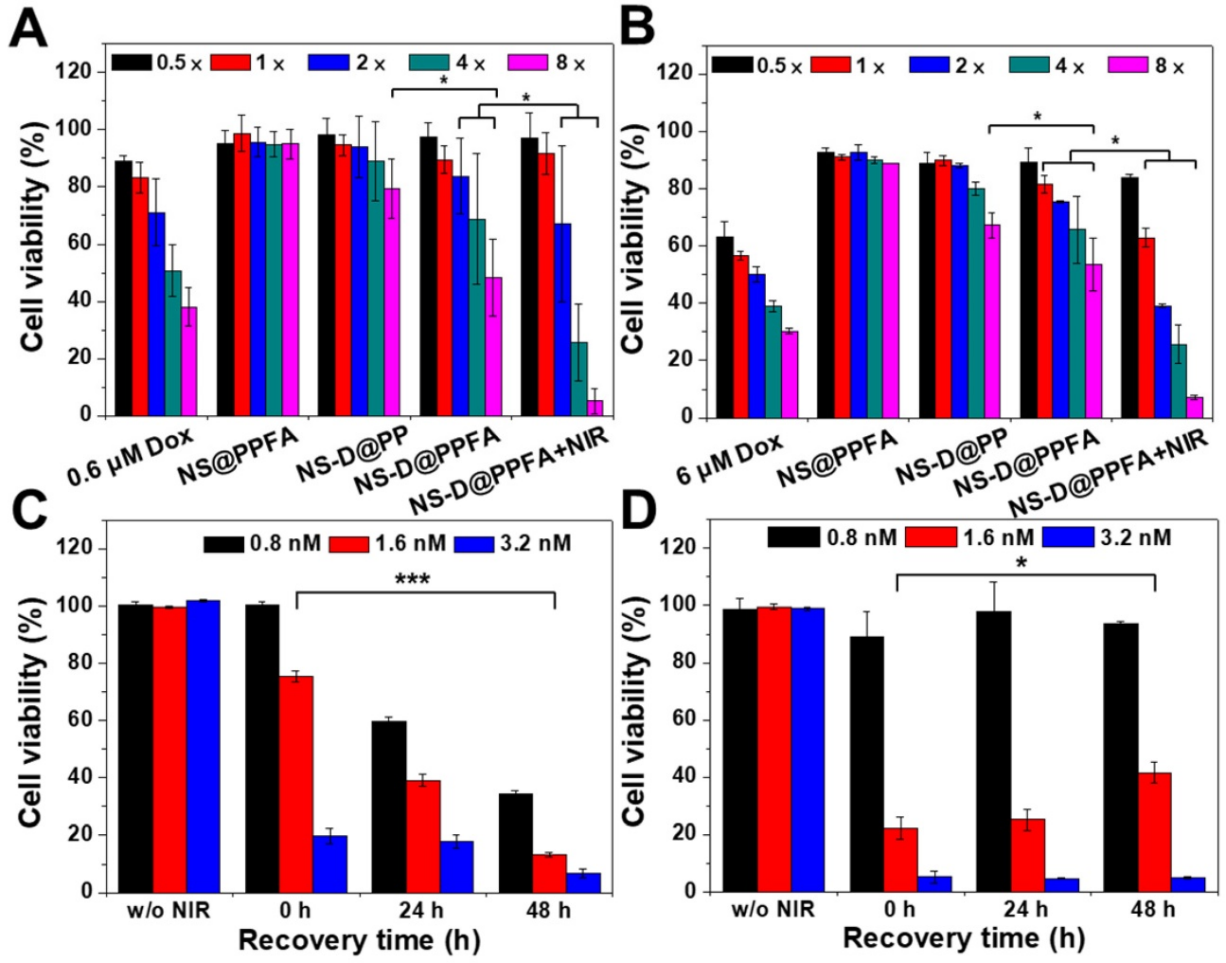
Cytotoxicity assay in (A) MCF-7 and (B) MCF-7/ADR cells. MCF-7 and MCF-7/ADR cells were incubated with different therapeutic nanoagents (1× = 0.4 nM NS) for 12 h and 24 h, respectively. After washing, cells were irradiated with NIR laser (3.6 W/cm^2^) for 10 min and recovered in fresh medium for additional 24 h. Viability response of MCF-7 and MCF-7/ADR cells was also examined at serial concentrations of DOX (0.3 - 4.8 μM and 3.0 - 48 μM, respectively). In addition, cell viability of MCF-7 cells were evaluated at different recovery period (0 - 48 h) after 6 h-exposure of (C) NS-D@PPFA and (D) NS@PPFA, respectively. Laser treatment was performed after removing excess nanoagents at energy density of (C) 3.6 W/cm^2^ for 10 min and (D) 0.9 W/cm^2^ for 3 min, respectively. *p < 0.05, ***p < 0.001.

**Cytotoxic activity of NS-D@PPFA toward MCF-7 cells.** The in vitro therapeutic effect of the developed nanoagents was examined using the AlamarBlue assay in MCF-7 cells. As shown in **Figure 4A**, NS-D@PPFA revealed a significant antitumor activity toward cancer cells compared with its payload-free (NS@PPFA) and nontargeted counterpart (NS-D@PP), respectively. The highest cytotoxicity was observed for NS-D@PPFA after NIR irradiation, indicating that the local photothermal action can lead to a combined effect of enhanced drug release and simultaneous chemo-PTT. Annexin-V and PI staining also exhibited a higher apoptosis level (51%) in this group than that of NS@PPFA (4%) and NS-D@PPFA (38%), respectively (**Figure S10**). Moreover, drug-resistant breast MCF-7/ADR cancer cells (DOX, IC50 = 12 μM) were also evaluated (**Figure 4B**). Similar to parental MCF-7 cells (DOX, IC50 = 2.4 μM), the highest cytotoxicity was observed for combined chemo-PTT in drug-resistant breast MCF-7/ADR cancer cells. The IC50 ratio (MCF-7/ADR to MCF-7) of irradiated NS-D@PPFA was approximately seven times lower than that of the conventional DOX treatment, suggesting that the developed nanoplatform with simultaneous dual therapeutic actions is beneficial for the treatment of drug-resistant cancer cells.

To further illustrate the advantage of combined therapy compared with that of PTT alone, the cell viability of MCF-7 cells was investigated at different recovery periods after 6 h of incubation with NS-D@PPFA (**Figure 4C**) and NS@PPFA (**Figure 4D**), respectively. In response to NIR irradiation, immediate photothermal damage was found in cells treated with both NS-D@PPFA and NS@PPFA in a dose-dependent manner. The results of the live/dead assay also revealed the maximum reduction in total cell counts at the highest dose (**Figure S11**). Following extended recovery, the population of cells that underwent combined treatment continued to decrease, whereas cells that underwent monotherapy survived (**Figure 4C and D**). This result suggests that the cytotoxic action exerted through the subsequent drug release after laser irradiation appears to be potent and long lasting, resulting in an effective and persistent antitumor activity.^1^ We also noted that the laser power required for the photothermal ablation of NS@PPFA-treated cells was lower than that required for the photothermal ablation of NS-D@PPFA-treated cells. This finding can be ascribed to the less efficient cellular uptake of NS-D@PPFA compared with NS@PPFA by MCF-7 cells, as measured using ICP-MS (**Figure S12**). A similar phenomenon of decreased cytotoxicity and cellular uptake was observed for MCF-7 cells coincubated with NS@PPFA and DOX (data not shown), indicating that the anthracycline drug showed some degree of inhibition on folate receptor-mediated endocytosis. The strong interaction between FA and DOX may lead to a less accurate spatial orientation for efficient binding with the tumor cell membrane.^48^

The selectivity of the developed nanoagent was examined in folate receptor-negative normal cells (NIH/3T3 and HaCaT cells).^49^, ^50^ As shown in **Figure S13**, the cellular toxicity of NS-D@PPFA against NIH/3T3 and HaCaT cells was not significantly different from that of NS-D@P (p = 0.7-0.8). In addition, the combined therapeutic effect of NS-D@PPFA on normal cells was less obvious than that on cancer cells, except for normal cells treated with the highest dosage. The results of the quantitative analysis performed using ICP-MS also agreed with the aforementioned finding; that is, the amount of intracellular Au detected in MCF-7 and NIH/3T3 cells treated with NS-D@PPFA (0.8 nM) was 22.4 and 7.9 pg/cell, respectively. The observation of a more preferential cellular uptake and cytotoxic effect of NS-D@PPFA on cancer cells than on normal cells indicates great potential to prevent the undesired systemic side effects of cancer treatment.

**Cytotoxic activity of NS-D@PPFA toward human umbilical vein endothelial cells (HUVECs).** In antiangiogenic therapy for cancer, the tumor vasculature is the primary target. The damage of vascular ECs can reduce the vascular density, leading to the inhibition of tumor growth by depriving the supply of oxygen and nutrients.^17^ In this study, the cytotoxicity of HUVECs exposed to NS-D@PPFA was investigated using an AlamarBlue assay. As shown in **Figure S14**, the cytotoxicity of NS-D@PPFA was similar to that of NS-D@PP; this finding could be attributed to the lack of the folate receptor on HUVECs.^51^ However, cells that underwent combined chemo-photothermal treatment exhibited significant cell injury, possibly due to the lower DOX tolerance of HUVECs (IC50 = 0.9 μM) than of MCF-7 (IC50 = 2.4 μM) and NIH/3T3 cells (IC50 > 5.0 μM).^52^, ^53^ This finding suggests that NS-D@PPFA exerts an antivascular effect through EPR-based accumulation and damage to ECs.

**Figure 5:**
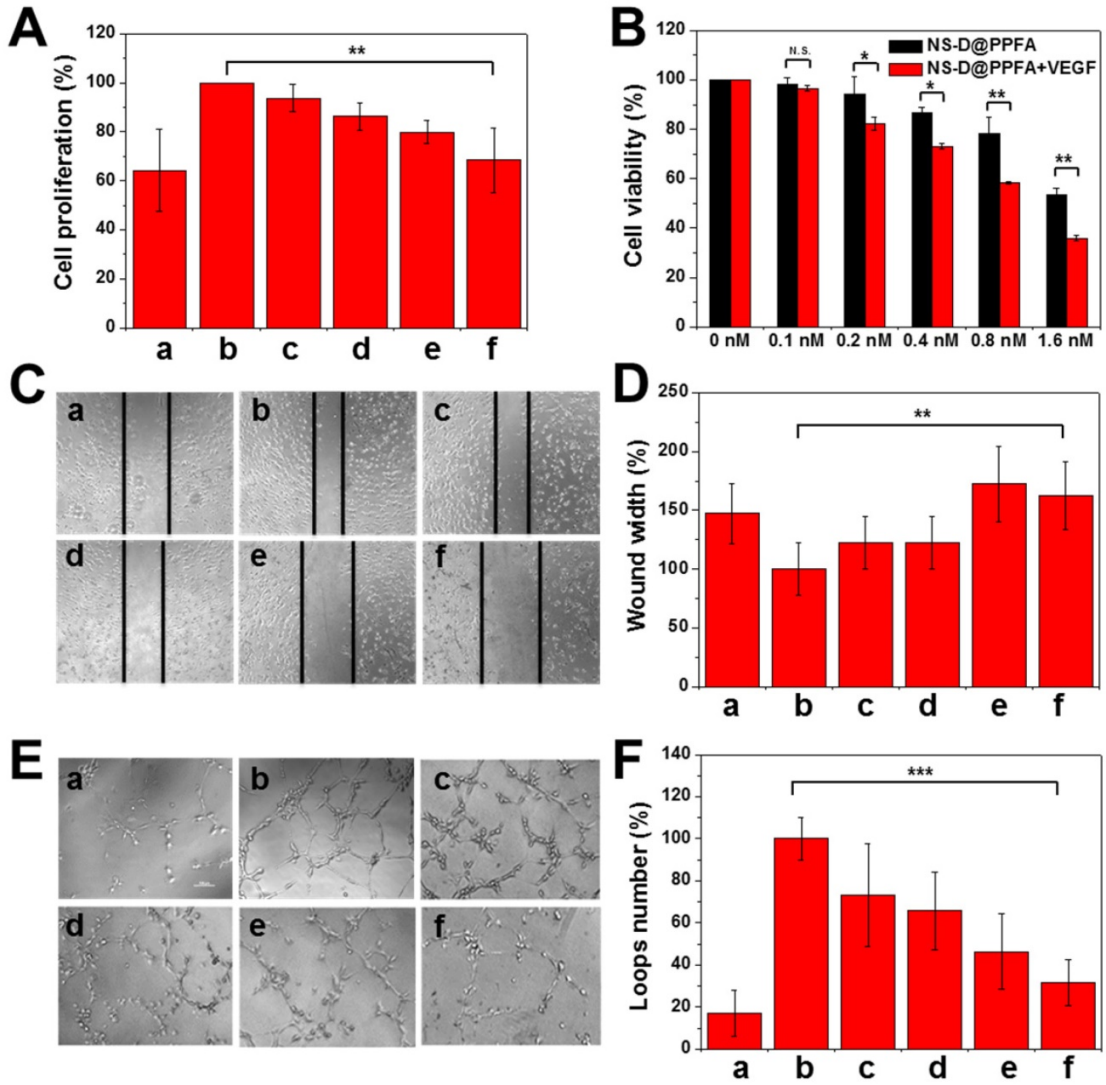
Inhibition of VEGF-induced HUVEC proliferation, migration and tube formation by various concentration of NS@PPFA. (A) The response of (a) non-stimulated and (b - f) 125 pM VEGF-stimulated cells to 48 h-exposure of (a, b) 0 (c) 0.1 (d) 0.2 (e) 0.4 (f) 0.8 nM NS@PPFA, respectively. The cell proliferation in group (b) was set as 100%. (B) Cell viability of HUVECs exposed to serial concentrations (0 - 1.6 nM) of NS-D@PPFA for 24 h in the presence or absence of VEGF (125 pM), respectively. The cell viability in each group without NS-D@PPFA treatment was set as 100%. (C - D) Representative microscopic images of HUVECs in migration assay (scale bar: 200 μm). Wound width was calculated as the average distance between the edges of the scratch. (E - F) Representative microscopic images of HUVECs in tube formation assay (scale bar: 100 μm). The number of the closed loop was determined within the image. The response of (a) non-stimulated and (b - f) 250 pM VEGF-stimulated cells to (a, b) 0 (c) 0.1 (d) 0.2 (e) 0.4 (f) 0.8 nM NS@PPFA was determined at (C - D) 6 h and (E - F) 12 h after dosing, respectively. The readings in group (b) were set as 100%, respectively. **p < 0.01, ***p < 0.001.

**Antiangiogenic effect of NS@PPFA on proliferation, migration, and tube formation of HUVECs.** In the angiogenesis process, VEGF-A165 is an essential growth factor that activates the VEGFR2 signaling pathway for subsequent tumor angiogenesis.^16^ Considering that PDA is a remarkable adhesive for different species, we evaluated whether the developed nanoagent can inhibit VEGF-induced proliferation of HUVECs similar to the aforementioned nanoparticles.^18^, ^19^ Payload-free NS@PPFA was first examined to prevent any additional cytotoxicity induced by DOX cargos. As shown in **Figure 5A**, enhanced EC proliferation was observed in response to incubation with VEGF-A165 (125 pM) for 48 h. However, the proliferation of HUVECs decreased significantly under the coexistence of NS@PPFA; an inhibition rate of approximately 89% was observed for the highest dosage (0.8 nM, cell proliferation = 68.3% ± 13.1%) compared with nonstimulated control (cell proliferation = 64.3% ± 16.6%). Furthermore, the antiproliferative effect of NS-D@PPFA on HUVECs was investigated in the presence or absence of exogenous VEGF-A165. As shown in **Figure 5B**, the lowest cell viability was observed in the presence of exogenous VEGF-A165. Compared with NS@PPFA (**Figure 5A**), NS-D@PPFA more effectively inhibited EC growth (0.8 nM, cell proliferation = 58.3% ± 0.6%), indicating that the simultaneous anti-VEGF and anticell proliferative potential of NS-D@PPFA make it a highly efficient nanotool in antiangiogenic therapy.

In addition to cell proliferation, cell migration and tube formation of HUVECs were assessed in vitro. According to microscopic images and quantitative analysis results (**Figure 5C-F**), a significant increase in EC migration (p = 0.006) and tube formation (p < 0.001) was observed for HUVECs after VEGF-A165 treatment (250 pM) compared with the control group. However, when coincubated with NS@PPFA (0.8 nM), the migration distance and number of loops significantly decreased with 100% and 80% of inhibition, respectively. This finding suggests that NS@PPFA can repress VEGF-induced cell migration and tube formation. In addition, we found that NS@PPFA at a test concentration of 0.2-3.2 nM (**Figure S14**) demonstrated negligible toxicity toward HUVECs. This result further confirmed that the antiangiogenic behavior of NS@PPFA is dependent on VEGF.

**Binding affinity of VEGF with NS@PPFA.** The mechanism underlying the anti-VEGF activity of NS@PPFA was investigated using a saturation binding assay. The concentration of NS@PPFA was kept constant at 4 pM, whereas the concentration of VEGF-A165 was gradient-diluted through overnight incubation in PBS (pH 7.4, 10% FBS). As shown in **Figure S15A and B**, approximately 75% of the feeding VEGF-A165 (1 nM) was bound to NS@PPFA, and the calculated *K*_d_ value was 2.68 × 10^-10^ M, which was approximately 100- to 1000-fold higher than that of the control protein (BSA, *K*_d_ = 1.99 × 10^-8^ M). These results suggest that NS@PPFA exhibits a strong binding affinity toward VEGF-A165, thereby outcompeting its capacity to VEGF targeting even in the presence of high-abundance plasma proteins (10% FBS). Moreover, VEGF-A165 is a basic protein with a high isoelectric point (pI ~ 8.5), making it positively charged in the physiological environment. It also contains two major binding domains with a greater number of positively charged residues are identified in the HBD domain.^19^ This finding further suggests a preferential binding of negatively charged nanomaterials, such as GO, through an electrostatic interaction with the HBD of VEGF-A165. Therefore, the interaction between NS@PPFA and VEGF-A165 was investigated in PBS at various pH values (10% FBS, pH 4.0, 7.4, and 9.8). As shown in **Figure S15C**, NS@PPFA exhibited superior adsorption ability at pH 7.4 compared with that at pH 9.8 and 4.0. VEGF-A165 became less positively charged in basic solution, and NS@PPFA exhibited less negative charges under acidic conditions (-8 mV) than at the neutral pH (-20 mV). Electrostatic interactions between NS@PPFA and VEGF-A165, particularly the HBD are considered crucial to their binding process; while simultaneously, additional attractive forces such as hydrogen bonding, hydrophobic interaction and π-π stacking may also be present.

**Figure 6:**
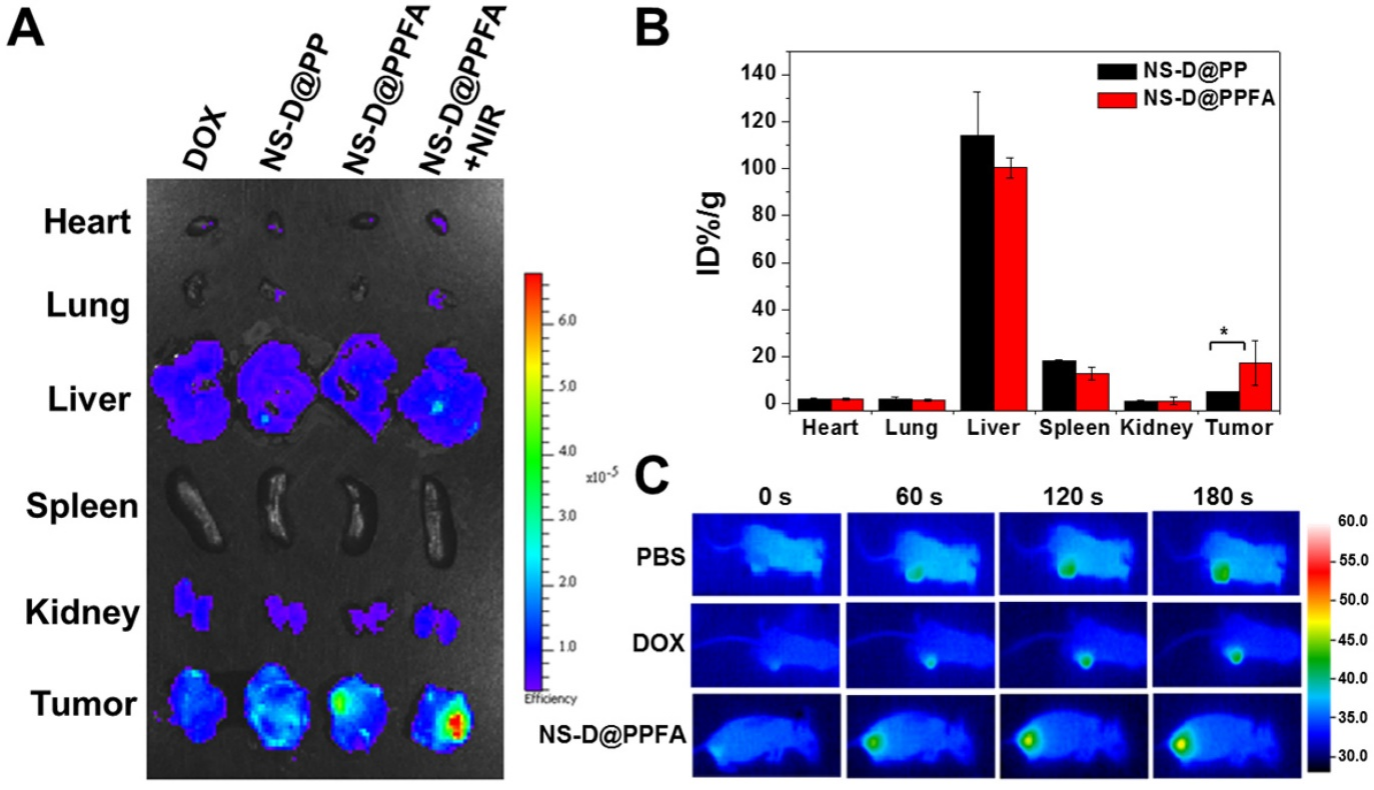
(A) Representative ex vivo fluorescent images of DOX in different organs and tumors following intravenous injection of mice with free DOX (5 mg/kg), NS-D@PP, NS-D@PPFA and NS-D@PPFA with NIR irradiation (6 mg/kg Au, 1.8 mg/kg DOX), respectively. Laser treatment (808 nm, 0.9 W/cm^2^, 3 min, 3 times) was performed twice at 24 and 48 h post-injection. Treated mice were scarified at 4 h after the 2nd irradiation, tumor and five organs (kidney, heart, spleen, lung, liver) were obtained, fixed with 4% paraformaldehyde, and imaged by IVIS (excitation/emission = 500/620 nm). (B) Percentage of injected dose per gram of tissue (% ID/g) of NS-D@PP and NS-D@PPFA (6 mg/kg Au) accumulated in different organs and tumors was quantified by ICP-MS at 52 h post-injection. (C) Representative photothermal images of tumor-bearing mice under different treatment (PBS, DOX and NS-D@PPFA) were recorded during the 2nd irradiation. *p < 0.05.

**In vivo biodistribution and photothermal effects.** To evaluate the tumor targeting and biodistribution of NS-D@PPFA in vivo, MCF-7/ADR tumor-bearing nude mice were intravenously injected with various nanocomposites (6 mg/kg Au, 1.8 mg/kg DOX) and free DOX (5 mg/kg), respectively. At 52 h post administration, the intrinsic fluorescence of DOX from the excised tumor and organs was detected using an ex vivo IVIS imaging system. As shown in** Figure 6A**, the highest fluorescence intensity was observed in the tumor obtained from NS-D@PPFA-treated mice, indicating that improved drug accumulation was achieved by both passive and active tumor-targeting mechanisms. A significant Au uptake in the tumor (up to 17% ID/g in **Figure 6B**) was also found in mice injected with NS-D@PPFA compared with its nontargeted counterpart (approximately 5% ID/g), further suggesting that folate receptor-mediated targeting can minimize tumor drug resistance by enhancing cellular accumulation and retention.^54^ Similar to previous findings, a significant fraction of Au could be detected in the liver (100% ± 4% ID/g) and spleen (13% ± 3% ID/g), whereas the remaining organs (heart, lung, and kidney) exhibited minimal Au accumulation.^55^-^57^ On the other hand, the fluorescence signal of NS-D@PPFA in the tumor site of treated mice remarkably increased after NIR irradiation (808 nm, 0.9 W/cm^2^, 3 min, 3 times; **Figure 6A**). A rapid temperature increase to approximately 48.0 °C also appeared accordingly (**Figure 6C**) compared with that of PBS or DOX control (40.9 and 41.7 °C, respectively), suggesting that the photothermal heating of NS-D@PPFA induced by light exposure is capable of on-demand drug release for subsequent tumor growth inhibition. This finding is consistent with the immunohistochemical results in **Figure S16**, which show that a pronounced increase in the HSP70 level^56^, ^57^ (p < 0.001) was observed in tumors of NS-D@PPFA-treated mice, followed by NIR irradiation, leading to an enhanced DOX signal in response to immediate photothermal stress.

**Figure 7:**
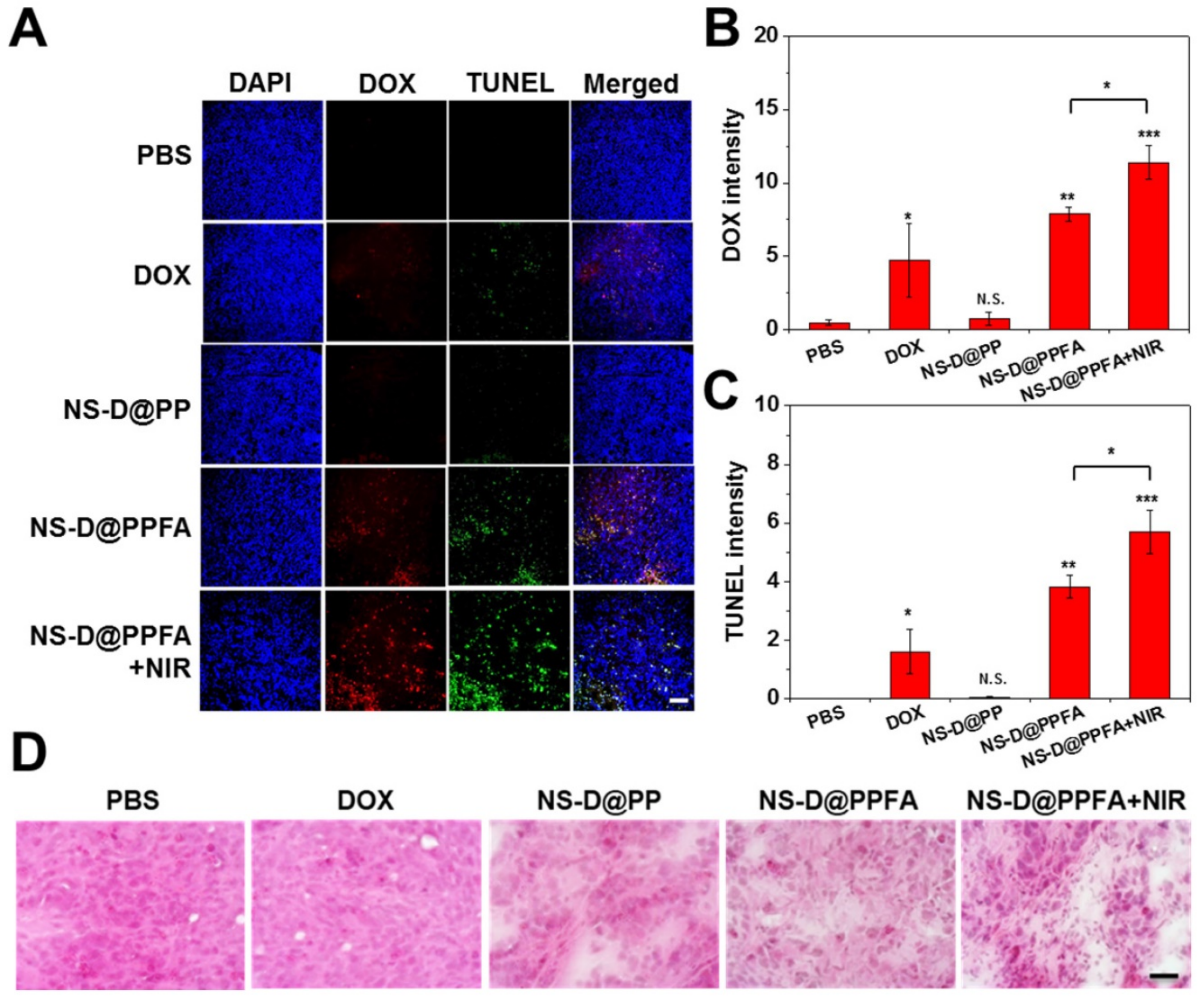
(A - C) Fluorescence (DOX and TUNEL) and (D) H&E staining microscopic images of tumor tissue sections harvested from MCF-7/ADR tumor-bearing nude mice injected intravenously with PBS, DOX (5 mg/kg), NS-D@PP, NS-D@PPFA and NS-D@PPFA with NIR irradiation (6 mg/kg Au, 1.8 mg/kg DOX), respectively. Laser treatment (808 nm, 0.9 W/cm^2^, 3 min, 3 times) was performed twice at 24 and 48 h post-injection. Images of the tumor sections were acquired at 4 h after the 2nd irradiation (scale bar: 50 µm). Quantification of the fluorescence intensity of (B) DOX and (C) TUNEL in microscopic images of resistant breast cancer tissue using ImageJ software. Mean fluorescence within a region of interest (ROI) was measured and normalized by DAPI fluorescence intensity. Data represented as mean ± SD, n=3. p* < 0.05, p** < 0.01, p*** < 0.001 versus PBS control.

**In vivo chemo-photothermal actions.** The antitumor efficacy of NS-D@PPFA in vivo was investigated by performing histological analyses. As shown in **Figure 7A and B**, the DOX signal was significantly higher in frozen tumor sections obtained from mice exposed to NS-D@PPFA than in those obtained from mice exposed to nontargeted NS-D@PP (p < 0.01). When subjected to NIR irradiation, the DOX signal of NS-D@PPFA became more intense (p < 0.05). A noticeable increase in necrotic and apoptotic cells visualized using TUNEL staining (p < 0.05 in **Figure 7C**) indicated that the combined chemo-photothermal action of the developed nanoagent enabled a superior antitumor activity. By contrast, a less obvious fluorescence signal was detected in the tumors of mice that received free DOX, possibly because the related toxicity was insufficient to induce desirable therapeutic outcomes. The corresponding H&E staining of tumor sections (**Figure 7D**) also demonstrated that NIR-illuminated NS-D@PPFA caused the most severe tissue damage, with the resultant appearances of swollen cell nuclei, ambiguous and vague intercellular gaps, and atrophic tumor structures being more evident compared with those in other groups.

**Figure 8:**
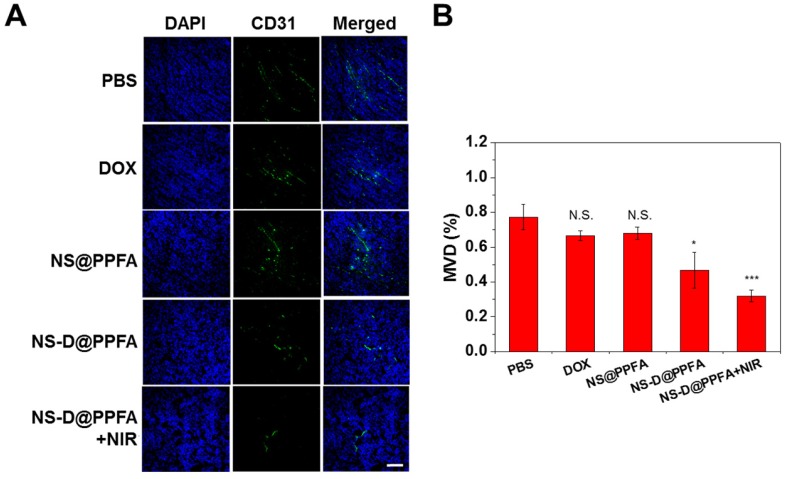
(A) The immunofluorescence CD31 staining of tumors after administration with PBS, DOX (5 mg/kg), NS@PPFA, NS-D@PPFA and NS-D@PPFA with NIR irradiation (6 mg/kg Au, 1.8 mg/kg DOX), respectively. Laser treatment (808 nm, 0.9 W/cm^2^, 3 min, 3 times) was performed twice at 24 and 48 h post-injection. Images of the tumor sections were acquired at 4 h after the 2nd irradiation (scale bar: 50 µm). (B) Evaluating the microvessel density (MVD, %) of resistant breast tumor xenografts after different treatments. p* < 0.05, p*** < 0.001 versus PBS control.

**In vivo antiangiogenic activities.** To determine whether NS-D@PPFA exerts an antiangiogenic effect on the tumor neovasculature, an immunohistochemical assay was performed to detect the microvessel density (MVD). CD31 (green) was used as an EC marker to represent the sites of tumor blood vessels.^60^ As shown in **Figure 8**, many vessels were observed in the saline control group, whereas low CD31-positive signals were detected after treatment with NS-D@PPFA (p < 0.05). The illuminated NS-D@PPFA caused the highest reduction in the tumor MVD (p < 0.001), further suggesting the involvement of combined chemo-PTT in tumor angiogenesis blood vessels. Expression of other angiogenic markers (VEGFR2 and pVEGFR2)^61^ was also immunohistochemically examined to elucidate the molecular mechanism of action of the developed therapeutic nanoagent. A similar result was observed for VEGFR2-positive signals (**Figure S17**), showing a microvessel inhibition of respectively 59% and 71% (p < 0.05) after treatment with NS-D@PPFA and illuminated NS-D@PPFA versus the saline control. However, the expression level of pVEGFR2 decreased gradually after treatment with NS@PPFA, NS-D@PPFA, and illuminated NS-D@PPFA. The extent of phosphorylation (pVEGFR2/VEGFR2 ratio) was nearly indistinguishable among the three groups. This finding indicates that the developed nanoagent might cause a moderate decrease in tumor vascularization along with a decreased activation level of VEGFR2 because of the successful trapping of VEGF that inhibits subsequent signaling events (angiogenesis process). In combination with the direct cytotoxic activity against the tumor vasculature, illuminated NS-D@PPFA can be the most effective antiangiogenic therapy, as characterized by the apparent loss of CD31 expression (i.e., vascularization) in **Figure 8**.

**Figure 9:**
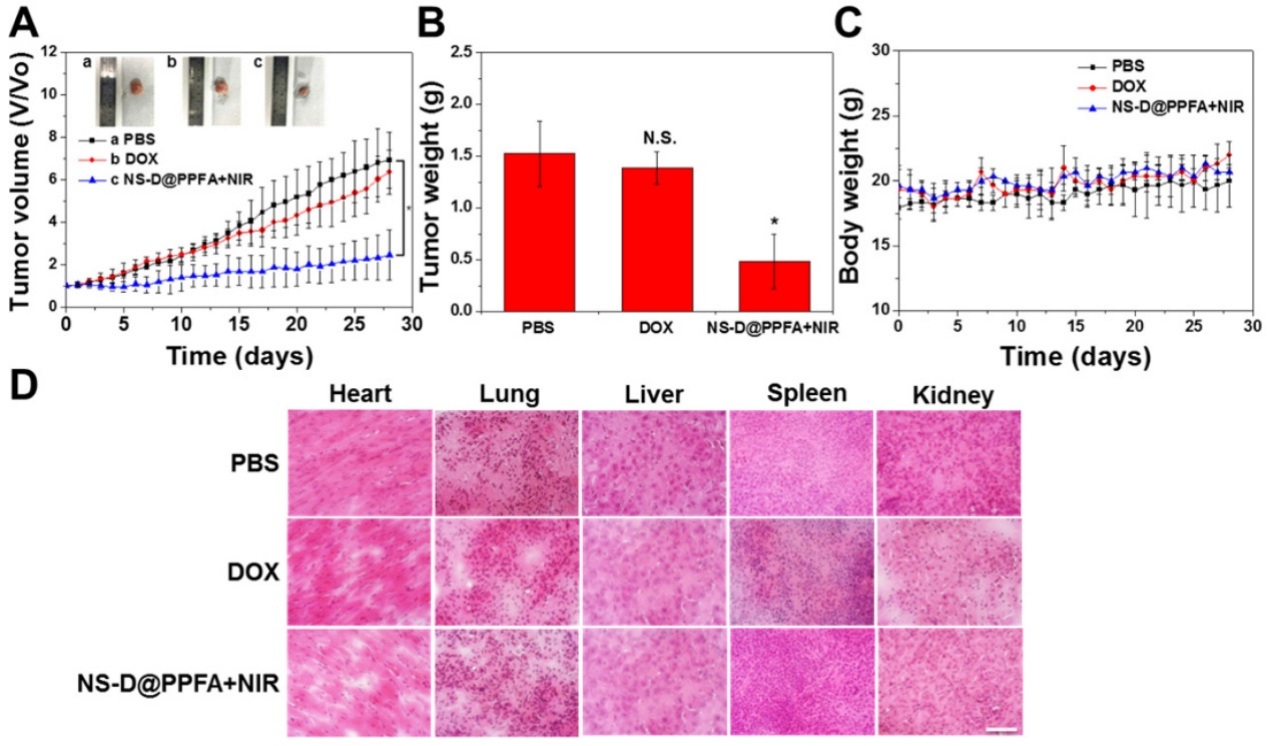
Antitumor efficacy of illuminated NS-D@PPFA on MCF-7/ADR tumor-bearing mice. (A) Tumor growth curve of mice receiving intravenously administration of PBS, DOX (5 mg/kg), and NS-D@PPFA (6 mg/kg Au; 1.8 mg/kg DOX) at day 0 and day 14, respectively. Tumors were treated with NIR irradiation (0.9 W/cm^2^, 3 min, 3 times) at 24 h and 48 h after each injection. (B) Tumor weight was measured on excised tumors at day 28 after different treatments. (C) Body weight monitoring of the treated mice over a period of 28 days. Data represented as mean ± SD, n=3. p* < 0.05 versus PBS control. (D) H&E-stained slices of major organs tissue of the mice with different treatments (scale bar = 50 μm).

**In vivo combination therapy for drug-resistant tumors.** The combined chemo-photothermal and antiangiogenic effects exerted by illuminated NS-D@PPFA were further assessed by monitoring tumor growth (**Figure 9A and B**). In particular, MCF-7/ADR tumor-bearing nude mice were treated with PBS, free DOX (5 mg/kg), or NS-D@PPFA (6 mg/kg Au, 1.8 mg/kg DOX) plus NIR. Compared with the saline control group, the illuminated NS-D@PPFA group exhibited significantly slower tumor growth (p < 0.05 vs. PBS at 28 days post-injection). The finding of a more pronounced growth inhibitory effect on the tumor xenograft in mice treated with illuminated NS-D@PPFA than in mice treated with free DOX revealed that combination therapy based on NS-D@PPFA demonstrated the most efficient antitumor activity. In addition, no death and no obvious weight loss (**Figure 9C**) were observed in all the experimental groups throughout the period. No indications of tissue injury were found in the H&E-stained sections of five major organs (the heart, lungs, liver, spleen, and kidneys) obtained from mice sacrificed on day 28 after intravenous injection (**Figure 9D**). All these results demonstrate that NS-D@PPFA and free DOX did not cause serious toxicity and side effects in mice. Moreover, NS-D@PPFA, which exhibited both anticancer and antiangiogenic activities, caused superior tumor eradication at a lower drug dose, making it a promising therapeutic strategy for MDR cancer.

## Conclusions

In summary, we successfully developed a multifunctional nanoagent NS-D@PPFA for targeted combination therapy to overcome drug resistance in breast cancer. The PDA coating on spiky Au NP can be a new nanoplatform with markedly improved photothermal stability and drug-loading efficiency. The subsequent decoration with PEG-FA also enabled a more specific cellular uptake and cytotoxicity in folate receptor-positive MCF-7 and drug-resistant MCF-7/ADR cells than in folate receptor-negative noncancerous cells. In response to NIR irradiation, the production of photothermal heating could not only accelerate drug release but also eradicate cancer cells synergistically. After systemic administration, NS-D@PPFA led to superior drug localization in tumors and improved therapeutic outcomes compared with conventional chemotherapy in drug-resistant human breast tumor xenografts. More importantly, the developed nanoagent played a critical role in the blockade of VEGF-mediated angiogenesis. The PDA coating strategy significantly inhibited the proliferation, migration, and tube formation of HUVECs. Furthermore, the decreased expression levels of CD31 and pVEGFR2 in tumor xenograft tissues demonstrated the potential of illuminated NS-D@PPFA in antivascular tumor treatment. Collectively, our study sheds new light on a previously unrecognized, antiangiogenetic facet of mussel-inspired PDA adhesives and may lead to the development of new therapeutic strategies against cancer treatment.

## Materials and Methods

**Chemicals.** Hydrogen tetrachloroaurate (HAuCl_4_), dopamine hydrochloride, and Doxorubicin (DOX) were purchased from Sigma-Aldrich (St. Louis, MO). 4-(2-hydroxyethyl)-1-piperazineethanesulfonic acid (HEPES) and Tris(hydroxymethyl)aminomethane (Tris) were purchased from J. T. Baker (Center Valley, PA, USA). Thiol-PEG (HS-PEG, M.W. = 5000 Da) and folic acid-PEG-thiol (HS-PEG-FA, M.W. = 5000 Da) was purchased from JenKem Technology (Beijing, China) and Peng Sheng Biotechnology (Shanghai, China), respectively. Dulbecco's phosphate-buffered saline (PBS) was purchased from Biosource (Camarilllo, CA, USA). Fetal bovine serum (FBS), penicillin-streptomycin, and Dulbecco's modified Eagle medium (DMEM) were obtained from Gibco (Grand Island, NY, USA). Alexa Fluor 633 conjugate transferrin and matrigel matrix high concentration were acquired from Invitrogen (Carlsbad, CA, USA). FITC Annexin V/Dead Cell Apoptosis Kit was purchased from BD Biosciences (San Jose, CA, USA). AlamarBlue® was purchased from AbD Serotec (Oxford, UK). Vasculife Basal Medium was purchased from LifeLine Cell Technology (CA, USA). TUNEL Assay (Alexa Fluor 647) was purchased from Thermo Fisher (Massachusetts, USA). Tissue-Tek OCT cryo gel was purchased from Sakura Finetek (Torrance, CA, USA). Anti-CD31 primary antibody was purchased from Arigo (Taipei, Taiwan).

**Synthesis of Au NS.** Au NS was synthesis by HEPES reductive method.^29^ Briefly, 16 μM of HAuCl_4_ (25 μM) was added to 1000 μL HEPES (140 mM, pH 7.4). After mixing, solution was sat undisturbed for 1 h to crystallization. The suspension was centrifuged with 5000 rcf for 15 min to remove excess reagents and the resulting pellet was redispersed in deionized (DI) water (250 μL) to obtain 4× NS (1.6 nM) for the following experiment.

**Preparation of NS-D@P.** To prepare PDA-coated Au NS for drug encapsulation, DOX (5 mM, 10 μL) and HS-PEG (0.5 mM, 4 μL) were added to the as-prepared NS (1.6 nM, 200 μL). Subsequently, DA (1 mg/mL, 200 μL) was added to the mixing solution, and Tris-HCl (pH 8.5, 10 mM, 386 μL) was added to provide a mild alkaline condition for DA polymerization.^28^ After 2 h reaction in room temperature, the mixture was centrifuged with 5000 rcf for 15 min to remove excess agents. The pellet was washed once and resuspended in DI water (200 μL) to obtain 1.6 nM NS-D@P.

**HS-PEG-FA capping on NS-D@P.** Prior to modification, HS-PEG-FA was activated by TCEP (mole ratio of HS-PEG-FA to TCEP is 1 : 2) for 1 h. Then, the activated HS-PEG-FA (500 μM, 40 μL) was mixed with NS- D@P (1.6 nM, 200 μL). Tris-HCl (pH 8.5, 10 mM, 160 μL) was added to provide alkaline condition for Michael addition reaction. The solution was stirred for 24 h in room temperature. After reaction finished, NS-D@PPFA was washed twice by centrifugation (5000 rcf, 15 min). The final pellet was resuspended in 200 μL DI water to obtain 1.6 nM NS-D@PPFA. Non-targeted NS-D@PP was also constructed using the same protocol with HS-PEG.

**Characterization of different nanoagents.** The measurements of hydrodynamic size and zeta potential of the as-prepared nanoagents were carried out on a dynamic light scattering (DLS) analyzer (Malvern Instrument, United Kingdom). Optical properties were measured via UV-Vis spectroscopy (Varian, Palo Alto, CA, USA). The samples were diluted in DI water and sonicated before measurement. The surface morphology of different nanoagents were imaged by transmission electron microscopy (TEM, Hitachi, Tokyo, Japan). To prepare samples for TEM images, colloidal suspension was dropped onto a carbon-coated copper grid and dried before observation. The DOX loading efficiency, DOX LE (%) and DOX loading content, DOX LC (%) of different nanoagents was determined by measuring the concentration of un-encapsulated drugs remained in the supernatant. Samples were diluted in HCl (pH 2.0) and quantified by microplate reader (Tecan Infinite 200, Tecan Group AG, Basel, Switzerland) for emission at 600 nm (excitation: 480 nm). DOX LE (%) and LC (%) were calculated using below formula:

DOX LE (%) = Weight of DOX encapsulated in nanoagent/Weight of feeding DOX × 100%

DOX LC (%) = Weight of DOX encapsulated in nanoagent/ Weight of nanoagent × 100%

Long-Termed Stability of NS-D@PPFA. The long-termed stability of NS-D@PPFA was evaluated by dispersing the samples in DMEM (10% FBS) for 48 h. At each point of time, part of samples were diluted in DI water for DLS measurement.

**In vitro photothermal effect and photo-stability of NS-D@PPFA.** Photothermal effect of NS-D@PPFA was investigated under a serial concentration in DI water, followed by 808 nm light irradiation (CW diode laser, LSR808NL-2000) at 0.9 W/cm^2^. Temperature of the solution was recorded at each exposure time by thermocouple; the corresponding thermal image was observed by an infrared camera. DI water was used as a control group in this experiment. The photo-stability was examined by laser on-off cycles at 0.9 W/cm^2^ (6 min laser on and 6 min laser off per cycle). The absorbance of NS-D@PPFA after laser irradiation (3.6 W/cm^2^, 10 min) was evaluated by UV-Vis spectra.

**DOX release of NS-D@PPFA.** To investigate the laser triggered drug release, NS-D@PPFA (1.6 nM) was dispersed in 100 μL PBS (pH 7.4 and 5.0), followed by a laser on-off irradiation cycles (0.9 W/cm^2^, 10 min laser on and 30 min laser off per cycle). At each time point, sample was centrifuged (5000 rcf, 10 min) and the supernatant was collected for DOX quantification. The remaining pellet was resuspended in PBS for next irradiation cycle. To investigate drug release in different buffers, NS-D@PPFA (1.6 nM) was dispersed in 100 μL of PBS (pH 5.0), citrate buffer (140 mM Na_2_HPO_4_, 60 mM citrate acid, pH 5.0), GSH (10 mM in citrate buffer, pH 5.0) and lysosomal buffer (23.5 mg/mL L-cysteine, citrate buffer, pH 5.0). After irradiation by NIR laser (0.9 W/cm^2^) for 10 min, the sample solution was centrifuged and the supernatant was collected for DOX quantification.

**Cell cultures.** MCF-7, MCF-7/ADR, NIH/3T3, and HaCaT cells were routinely cultured in DMEM supplemented with 10% FBS and 1% antibiotic penicillin/streptomycin. HUVECs were cultured in endothelial cell growth medium (ECG). All of cells were cultivated under a humidified atmosphere with 5% CO_2_ - 95% air atmosphere at 37 °C.

**Cellular uptake and intracellular drug release of NS-D@PPFA.** To investigate the specificity of NS-D@PPFA toward cancer cells, MCF-7 cells were seeded on glass slices (5 × 10^4^ cells) for 24 h. After cell adhesion, the culture medium was replaced by 1% BSA containing washing buffer [4.5 g/L glucose and 5 mM MgCl_2_ in Dulbecco's PBS with calcium chloride and magnesium chloride (Sigma-Aldrich)]. 0.8 nM of NS-D@PP, NS-D@PPFA and NS-D@PPFA plus free FA (1 mM) was added for 2 h incubation, respectively. For microscopic imaging, cells were washed with PBS and stained with fluorescent transferrin (500 nM in 1% BSA containing PBS) at 37°C in a humidified atmosphere of 5% CO_2_ for 30 min. After removing excess reagents by rinsing with PBS, cells were fixed with 4% formaldehyde for 20 min at room temperature. Cells were then washed with PBS and cell nuclei were stained with DAPI (1 μM for 15 min). Afterwards, cells were mounted in ProLong® antifade reagent and examined with a fluorescence microscope (Olympus, Center Valley, PA, USA). The following data analysis was carried out using microscopic software (ZEM). To quantify the intracellular fluorescence signal of DOX, MCF-7 cells were seeded in 48-well plates (5 × 10^4^ cells) and incubated with different concentrations of nanoagents for 2 h. The cells were washed with PBS and collected using trypsinization. After centrifugation at 500 rcf for 5 min to remove residual proteins, cells were washed and resuspended with PBS for fluorescence measurement via flow cytometer (BD Bioscience, Franklin Lakes, NJ, USA). To determine the intracellular gold content, collected cells were digested with aqua regia overnight. The resulting samples were diluted with 2% HNO_3_ for inductively coupled plasma mass spectrometry (ICP-MS) analysis. To evaluate photo-triggered drug release in vitro, NS-D@PPFA-treated cells were rinsed with washing buffer, followed by NIR irradiation (0.9 W/cm^2^, 15 min). Exposed cells were stained with fluorescent transferrin and DAPI for fluorescence microscopic imaging. Trypsinized cells were subjected to flow cytometric analysis.

**In vitro cytotoxicity of NS-D@PPFA.** The cytotoxicity of NS@PPFA, NS-D@PP, NS-D@PPFA, NS-D@PPFA plus laser, and free DOX toward MCF-7 cells were evaluated using AlamarBlue assay. MCF-7 cells were seeded in a 96-well plate (6000 cells) for 24 h. After cells adhesion, the original culture medium was removed and replaced with fresh medium containing the above samples with various concentrations. After 12 h incubation, samples were removed and replaced by fresh medium. Laser treatment (808 nm, 3.6 W/cm^2^, 10 min) was performed for combination therapy. After 24 h of recovery, cells were treated with AlamarBlue@ reagent for 2 h. Fluorescence intensities at 590 nm (I_590_) were measured with the excitation at 540 nm. Cell cytotoxicity of NS-D@PPFA was also performed in MCF-7/ADR, NIH/3T3, HaCaT and HUVECs under the same condition except the drug exposure duration in MCF-7 resistant subline was extended to 24 h. Cell viability of MCF-7 cells was also detected at different recovery periods (0, 24, 48 h) after combined and single therapy. Cells were incubated with NS-D@PPFA and NS@PPFA for 6 h, followed by laser irradiation at 3.6 W/cm^2^ and 0.9 W/cm^2^ for 10 min and 3 min, respectively. Direct cellular damage induced by photothermal effect was also examined by calcein AM and Propidium Iodide (PI) staining (30 min) in fluorescence microscope.

**Cell apoptosis assay of NS-D@PPFA.** The assessment of apoptosis in response to different treatment was investigated using Annexin V and PI staining. Briefly, MCF-7 cells were seeded in a 48-well plate (2 × 10^4^ cells) for 24 h. Cells were incubated with different nanoagents for 24 h. After removing excess reagents, laser irradiation (0.9 W/cm^2^, 10 min) was performed for combination therapy. Cells were recovered for additional 24 h. Trypsinized cells were collected by centrifugation and resuspend in PBS for 15 min-incubation with Annexin V and PI. Analysis was carried out by flow cytometry.

**Adsorption of VEGF on NS@PPFA.** To evaluate VEGF adsorption on NS@PPFA, NS@PPFA (4 pM) was incubated with various concentrations of VEGF-A165 (0 - 8000 pM) in PBS (10% FBS, pH 7.4) overnight. After centrifugation, unbound VEGF remained in the supernatants was collected for VEGF quantification using VEGF ELISA assay (R&D Systems, Minneapolis, MN, USA). *K*_d_ was calculated by fitting the titration curve to the single site saturation binding equation [B_VEGF_]/ [Free-VEGF] = B_max_/*K*_d_ - [B_VEGF_]/*K*_d_ using SigmaPlot software. Where [B_VEGF_] is the concentration of bound VEGF, [Free-VEGF] is the concentration of unbound VEGF, B_max_ is the maximal number of binding sites. To conduct VEGF adsorption in various pH condition, NS@PPFA (4.0 and 0.4 pM) were incubated with 1000 pM VEGF in PBS (10% FBS, pH 4.0, 7.4, and 9.8, respectively) overnight. Unbound VEGF was removed and quantified by ELISA assay.

**Adsorption of BSA on NS@PPFA.** The adsorption of BSA on NS@PPFA was assessed as a control group. To quantify the adsorption of BSA on NS@PPFA, FITC labeled BSA was prepared. Briefly, 1 mL BSA (2 mg/mL) was mixed with 100 μL FITC (1 mg/mL) in 0.1 M Na_2_CO_3_ buffer (pH 9.0) for 8 h in the dark. The resulting solution were centrifuged (5000 rcf, 20 min) with microcon centrifugal filters (MWCO 50 kDa) to remove unbound FITC. FITC-BSA was collected and stored in PBS (pH 7.4) at -20 °C. For BSA adsorption, NS@PPFA (0.8 nM) was incubated with various concentration of FITC-BSA (0 - 800 nM) in PBS (10% FBS, pH 7.4) overnight. The resulting solution was centrifuged (5000 rcf, 15 min) and the supernatant was collected for fluorescence measurement (Ex: 480 nm, Em: 525 nm). The adsorption of BSA in various pH value was performed via mixing the NS@PPFA (800 and 80 pM) and FITC-BSA (200 nM) in PBS (10% FBS, pH 4.0, 7.4, and 9.8, respectively) overnight. After centrifugation, the supernatant was collected for fluorescence measurement.

**Proliferation and migration assay of HUVECs.** HUVECs were seeded into 48-well plates (6000 cells) for 24 h. The original medium was removed and replaced with various concentration of NS@PPFA (0 - 0.8 nM) in 125 pM VEGF-containing M199 medium (2% FBS). After 48 h incubation, the cell viability of HUVECs was detected using AlamarBlue assay. The effect of NS@PPFA on HUVEC migration was assessed by wound healing assay. Sterile cell culture inserts were placed in a 24-well plate. HUVECs (4 × 10^4^ cells) were seeded afterwards and incubated for 12 h. The inserts were then removed and the original medium was replaced by various concentration of NS@PPFA (0 - 0.8 nM) in 250 pM VEGF-containing M199 medium (2% FBS). The extent of cell migration to fill the empty area was evaluated by microscopic imaging at 12 h. Images of six randomly-selected positions in each well were acquired to calculate the average width of wound.

**Matrigel-based tube formation assay of HUVECs.** Prior to the experiment, matrigel was dissolve in 4 °C overnight. 114 μL Matrigel was added to 48-well plate and incubated for 30 min in 37 °C for polymerization. Afterwards, HUVECs (1.25 × 10^4^ cells) were seeded and treated with various concentration of NS@PPFA (0 - 0.8 nM) in 250 pM VEGF containing M199 medium (2% FBS) for 6 h. Tube formation images were captured with a digital microscope camera system. 10 images of randomly-selected positions were acquired in each well to calculate the average loop numbers.

**Xenograft tumor model and antitumor efficacy in vivo.** The animal experiment has been approved by the Institutional Animal Care and Use Committee of National Tsing Hua University, Taiwan. Five-week old BALB/c female nude mice were acclimatized for 2 weeks. To establish tumor xenograft, subcutaneous mouse injection of 1 × 10^7^ MCF-7/ADR cells in PBS (100 μL, contenting 50% matrix gel) was conducted on the right thigh and fed with β-estradiol at 8 mg/L in daily drinking water to induce the growth of breast tumor. The feeding of β-estradiol was stopped 1 week before the onset of treatment. When tumor grew to 200 mm^3^, MCF-7/ADR tumor-bearing mice were intravenously injected with PBS, DOX (5 mg/kg) and NS-D@PPFA (6 mg/kg Au, 1.8 mg/kg DOX) + NIR on day 1 and day 14, respectively. Irradiated groups were exposed by NIR laser (808 nm, 0.9 W/cm^2^, 3 min) 3 times with a time gap of 5 min, at 24 and 48 h post-injection, respectively. To evaluate the antitumor efficacy and side effect, tumor volume and body weight were measured every day. Tumor volume (V) was measured by digital caliper and calculated using the formula: V = W^2^ × L/2, where W is the short axis and L is the long axis. Relative tumor volume was calculated as V/V_0_, where V_0_ is the tumor volume before treatment. All the mice were sacrificed on day 28 and the major organs (heart, liver, spleen, lungs, kidneys, and tumor) were collected for further tissue sections.

**In vivo biodistribution.** When the average of tumor volume reached around 400 mm3, the mice were divided into 4 groups: DOX (5 mg/kg), NS-D@PP (6 mg/kg Au), NS-D@PPFA (6 mg/kg Au) and NS-D@PPFA (6 mg/kg Au) + NIR. After 24 h and 48 h post-intravenous injection, mice receiving NS-D@PPFA + NIR were exposed with 808 nm laser (0.9 W/cm^2^, 3 times for 3 min). Subsequently, all mice were sacrificed at 52 h post-injection; five organs (heart, liver, spleen, lung, and kidney) and tumors were harvested for ex vivo DOX imaging (IVIS, xenogeny, alameda, CA, USA). For ICP-MS analysis, tumor and organs (heart, liver, spleen, lung, and kidney) from mice receiving NS-D@PP and NS-D@PPFA were dissected, dried and weighed, followed by acid digestion and ICP-MS measurement. The corresponding data was expressed as the percentage of the injected dose per weight of tissue (%ID/g).

**Immunohistochemistry and histological Studies.** For hematocylin and eosin (H&E) staining, mice were euthanized at indicated time. Subsequently, tumors and organs were harvested and fixed with 10% paraformaldehyde. Tumors and organs were embedded in OCT, cryo-sectioned at 10 µm thickness and mounted onto slides. The sliced tissues were fixed in 100% methanol (-20 °C) and 10% paraformaldehyde for 10 min and 30 min, respectively. After the staining with H&E, tissue samples were covered with xylene-based mounting medium and examined under a light microscope (Olympus, Center Valley). For TUNEL assay, fixed tissue slides were washed by PBS and treated with proteinase K for 30 min. Slides were washed with PBS three more times (5 min each) and stained with Click-iT Plus TUNEL reaction cocktail for 30 min at 37 °C in the dark. Excess reagent was removed by washing twice each slide with PBS (3% BSA) for 5 min. Afterwards, the tissue sections were counterstained with DAPI covered with Prolong® Gold Antifade Reagent for fluorescence microscopic imaging. For heat shock protein (HSP) analysis, cryo-sliced tissues after paraformaldehyde fixation were washed twice with PBS (5 min each). HSP 70 primary antibody (1:50 dilution) was added and left to react overnight. Tumor slices were then washed twice with PBS (5 min each) and incubated with Cy5 labeled secondary antibody for 2 h in the dark. The sections were stained with DAPI and covered with Prolong® Gold Antifade Reagent for fluorescence microscopic imaging. For CD31, VEGFR2 and pVEGFR2 immunohistochemical staining, the fixed sections were washed and incubated with 0.1% Triton X-100 (containing 3% BSA) for 10 min to increase the permeability. The tissue sections were initially blocked by PBS containing 5% FBS and 0.3% Triton X-100 for 1 h. After blocking the non-specific sites, the slides were incubated with anti-CD31 or anti-VEGFR2, or anti-pVEGFR2 antibody in dilution buffer (1:50, 0.3% Triton X-100 and 1% BSA in PBS) overnight at 4 °C. After careful washing, slides were incubated with Alexa 488 fluorochrome-conjugate secondary antibody in dilution buffer (1:100) for 2 h in the dark. After immunohistochemical staining, slides were stained with DAPI and covered with Prolong® Gold Antifade Reagent. Images were examined by a fluorescence microscope. Microvessels density (MVD) were quantified using ImageJ software. The ratio of the pixel in signal area to the background pixels were measured to determine the vascular density.

## Figures and Tables

**Table 1:**
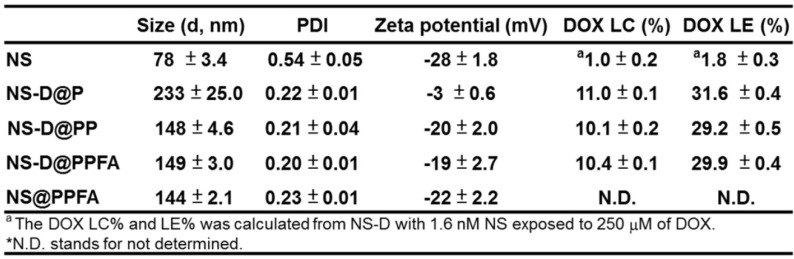
The characterization of different NS nanocomposites.

## Supplementary Material

Synthesis and characterization of the materials: UV-Vis, TEM images, hydrodynamic size, drug encapsulation and release analysis; photothermal evaluation; cellular uptake studies: flow cytometry, ICP-MS and fluorescence microscopic images; cytotoxicity, apoptosis and live/dead assays; VEGF binding test; In vivo immunohistochemical analysis. Figure S1-S17.
