# Supplementary material for: Polydopamine-coated gold nanostar for combined antitumor and antiangiogenic therapy in multidrug-resistant breast cancer
Source: Nanotheranostics. 2019 Jun 6;3(3):266–83. doi: 10.7150/ntno.36842 (PMC6584135; doi:10.7150/ntno.36842)
Supplement: Supplementary file 1 — Synthesis and characterization of the materials: UV-Vis, TEM images, hydrodynamic size, drug encapsulation and release analysis; photothermal evaluation; cellular uptake studies: flow cytometry, ICP-MS and fluorescence microscopic images; cytotoxicity, apoptosis and live/dead assays; VEGF binding test; In vivo immunohistochemical analysis. Figure S1-S17. [file ntnov03p0266s1.pdf]

Polydopamine-coated gold nanostar for combined antitumor and antiangiogenic therapy in multidrug-resistant breast cancer

You-Hong You[1], *Yu-Feng Lin*[1,2], *Bhanu Nirosha*[1], *Huan-Tsung Chang*[2] *and Yu-Fen Huang*[1,*]

[1] Department of Biomedical Engineering and Environmental Sciences, National Tsing Hua University, Hsinchu 30013, Taiwan, ROC

[2] Department of Chemistry, National Taiwan University, Taipei 10617, Taiwan, ROC

E-mail: yufen@mx.nthu.edu.tw

Supplementary Figures:

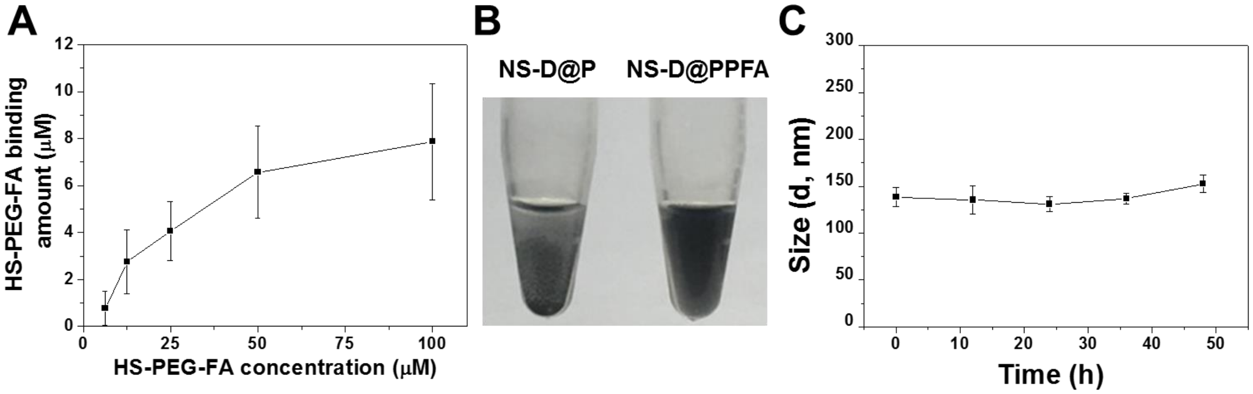

Figure S1. The modification of HS-PEG-FA on NS-D@P. (A) NS-D@P (1.6 nM) was exposed to serial concentrations of HS-PEG-FA. Quantitative analysis was performed by measuring the absorbance at 350 nm of the unbound HS-PEG-FA collected from the supernatants. (B) Photo-images of the aqueous suspensions of NS-D@P and NS-D@PPFA in PBS (pH 7.4) after 2 h incubation. (C) The colloidal stability of NS-D@PPFA in DMEM (10% FBS) was analyzed by dynamic light scattering (DLS) over 48 h.

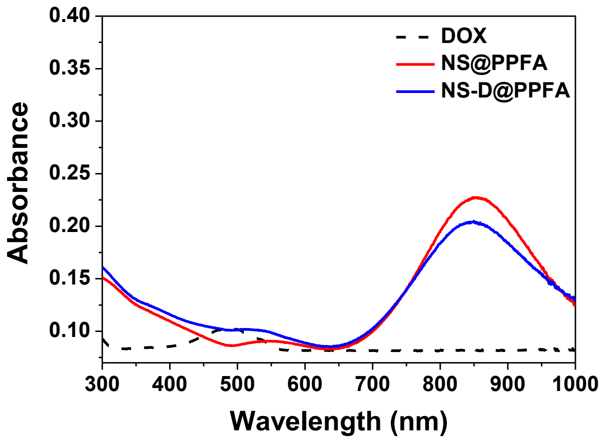

Figure S2. The UV-Vis spectra of NS@PPFA and NS-D@PPFA versus free DOX.

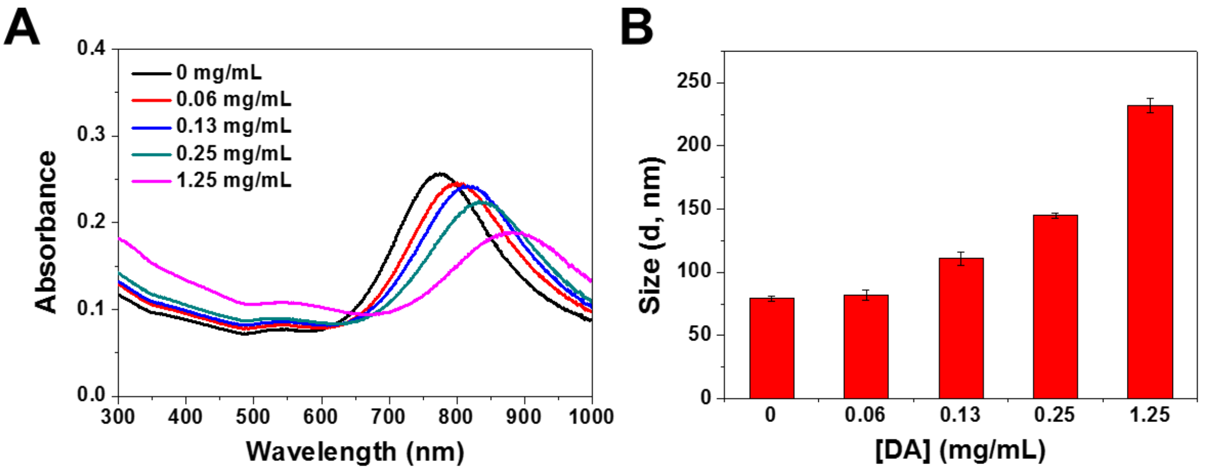

Figure S3. (A) UV-Vis spectra and (B) hydrodynamic size of NS-D@PPFA prepared with serial concentrations of DA (0 – 1.25 mg/mL).

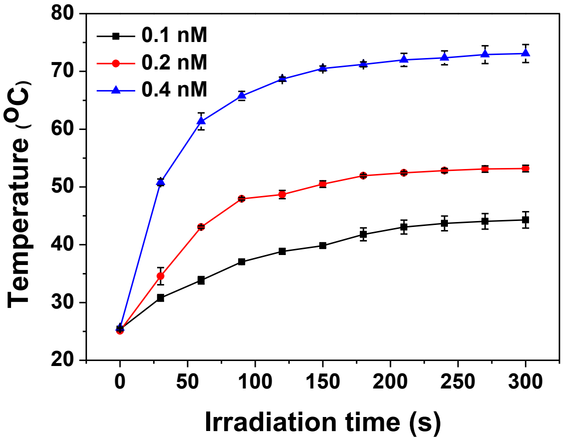

Figure S4. Photothermal effect by NS-D@PPFA in DI water. Temperature versus time profile for varying concentrations of NS-D@PPFA (0.1 – 0.4 nM) exposed to NIR laser (808 nm, 0.9 W/cm^2).

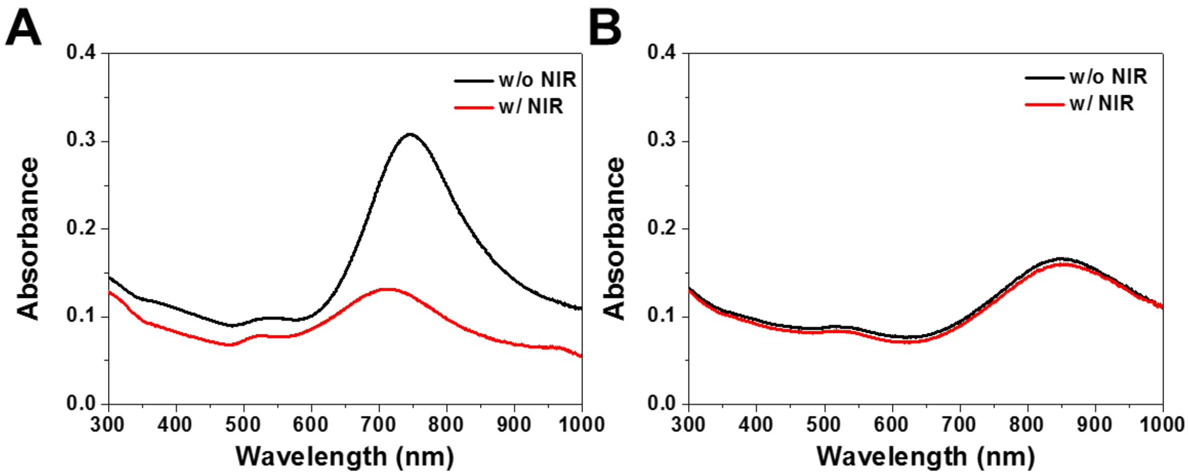

Figure S5. UV-Vis spectra of (A) bare Au NS and (B) NS-D@PPFA (1.6 nM) with or without NIR laser irradiation (3.6 W/cm^2, 10 min).

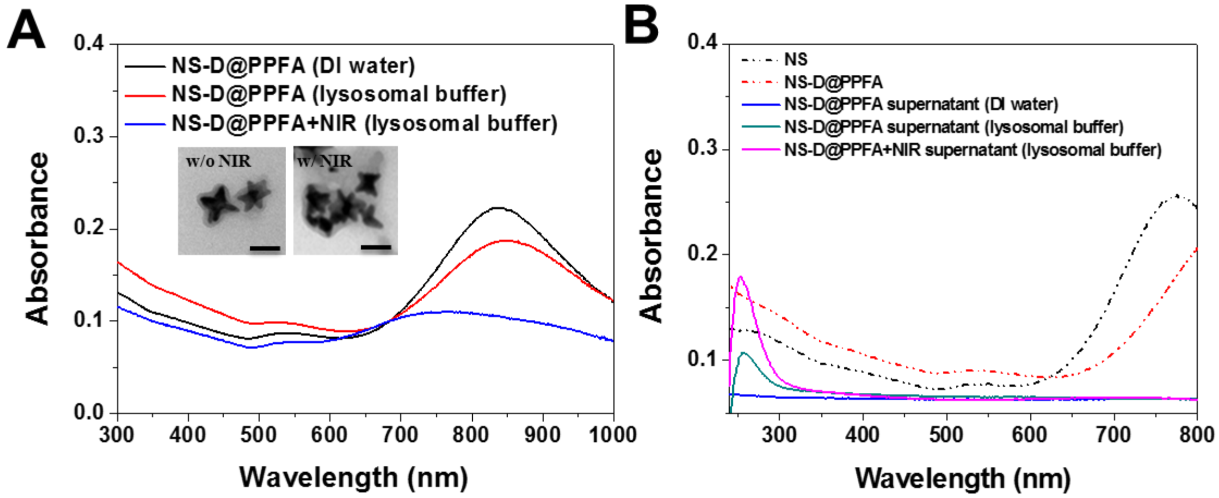

Figure S6. (A) UV-Vis spectra and TEM images (inset) of NS-D@PPFA (1.6 nM) suspended in lysosomal buffer (23.5 mg/mL L-cysteine in citrate buffer, pH 5.0) irradiated by NIR laser (2.0 W/cm^2, 20 min). Scale bar: 50 nm. (B) UV-Vis spectra of the supernatants (2-fold in concentration) collected from the samples under different treatments. The corresponding spectra for the control groups (NS and NS-D@PPFA in DI water) are displayed in dotted lines.

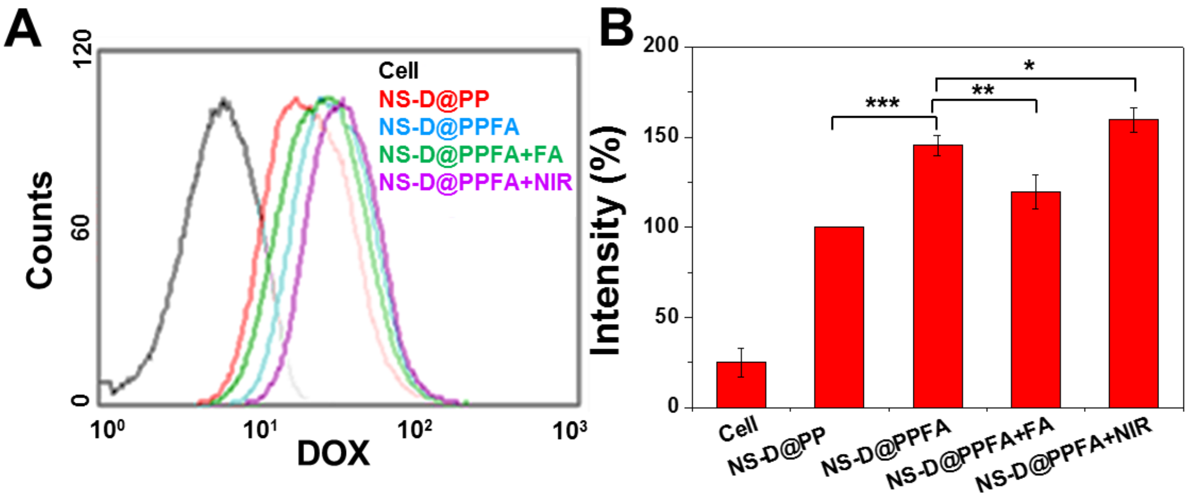

Figure S7. (A) Flow cytometric histogram and (B) quantitative analysis of MCF-7 cells after 2 h-incubation with NS-D@PP, NS-D@PPFA, NS-D@PPFA+FA, and NS-D@PPFA+NIR, respectively. The mean fluorescence intensity in ND-D@PP–treated cells was set as 100%. For competitive assay, cells were pre-treated with free FA for 1 h, followed by a subsequent incubation with NS-D@PPFA. For NIR exposure, cells treated with NS-D@PPFA were washed with fresh medium and irradiated with NIR laser (0.9 W/cm^2) for 15 min. *$p < 0.05$, **$p < 0.01$, ***$p < 0.001$.

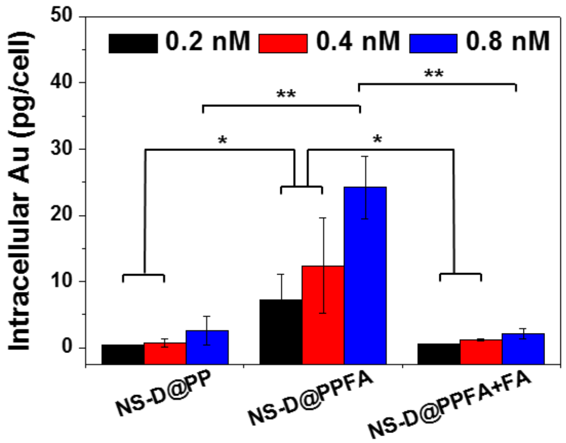

Figure S8. The quantitative analysis of intracellular Au in MCF-7 cells determined by ICP-MS. Cells were incubated with different nanoagents of serial concentrations (0.2 – 0.8 nM) for 2 h. *$p < 0.05$, **$p < 0.01$.

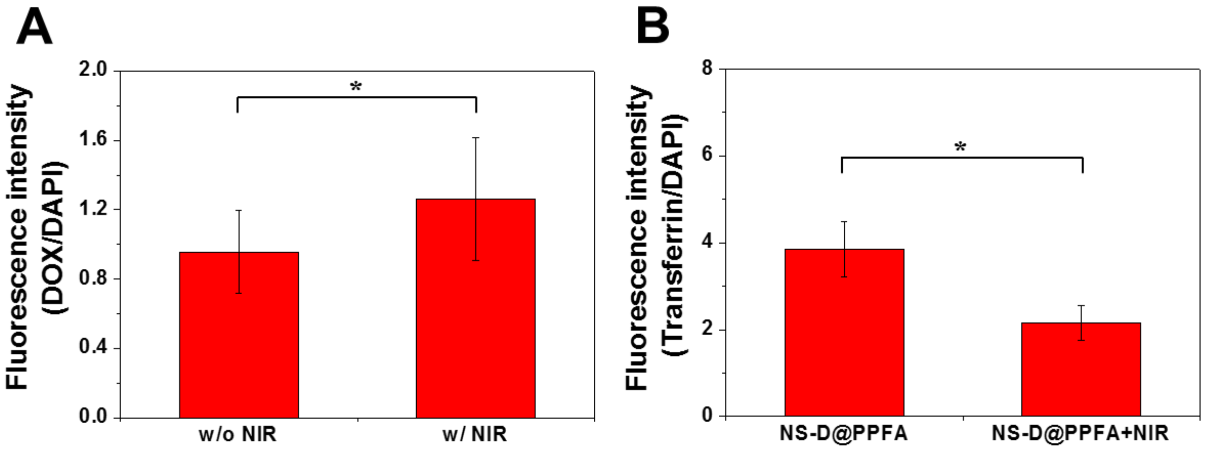

Figure S9. Quantitative analysis of NIR irradiation-induced endolysosomal escape and DOX release of NS-D@PPFA. The treatment condition was kept the same as in Figure 3. The fluorescence intensity of (A) DOX and (B) Alexa Fluor 633 conjugate transferrin in microscopic images of MCF-7 cells was quantified using ImageJ software. Mean fluorescence within a region of interest (ROI) was normalized by DAPI fluorescence intensity. $*p < 0.05$.

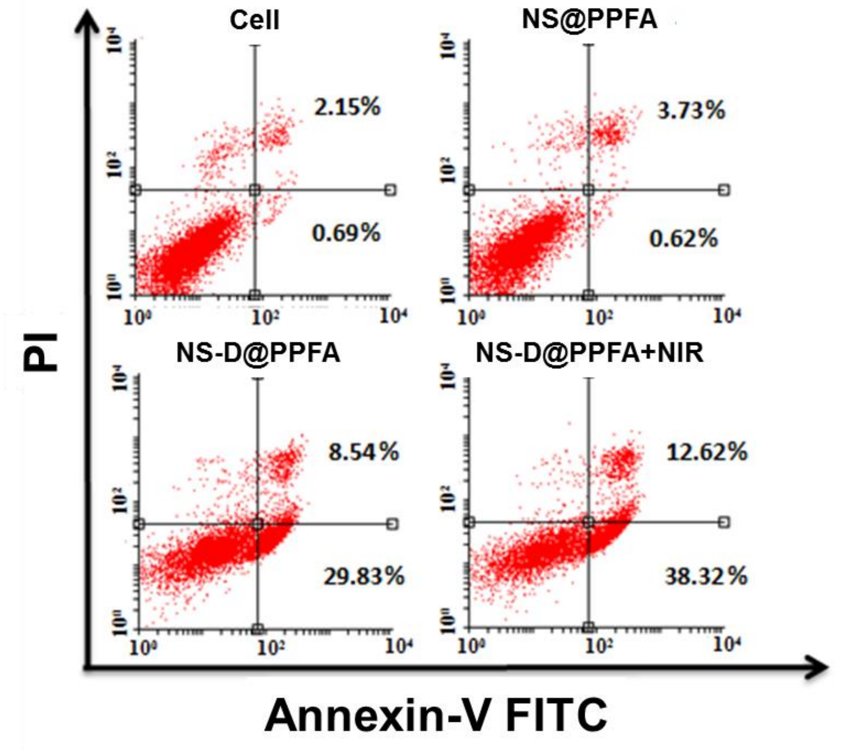

Figure S10. Cell apoptosis assay of NS@PPFA, NS-D@PPFA and NS-D@PPFA+NIR in MCF-7 cells by Annexin-V and PI double staining and flow cytometric measurement. Cells were incubated with different nanoagents (0.8 nM) for 24 h. After washing, cells were irradiated with NIR laser (0.9 W/cm^2) for 10 min and recovered in fresh medium for additional 24 h.

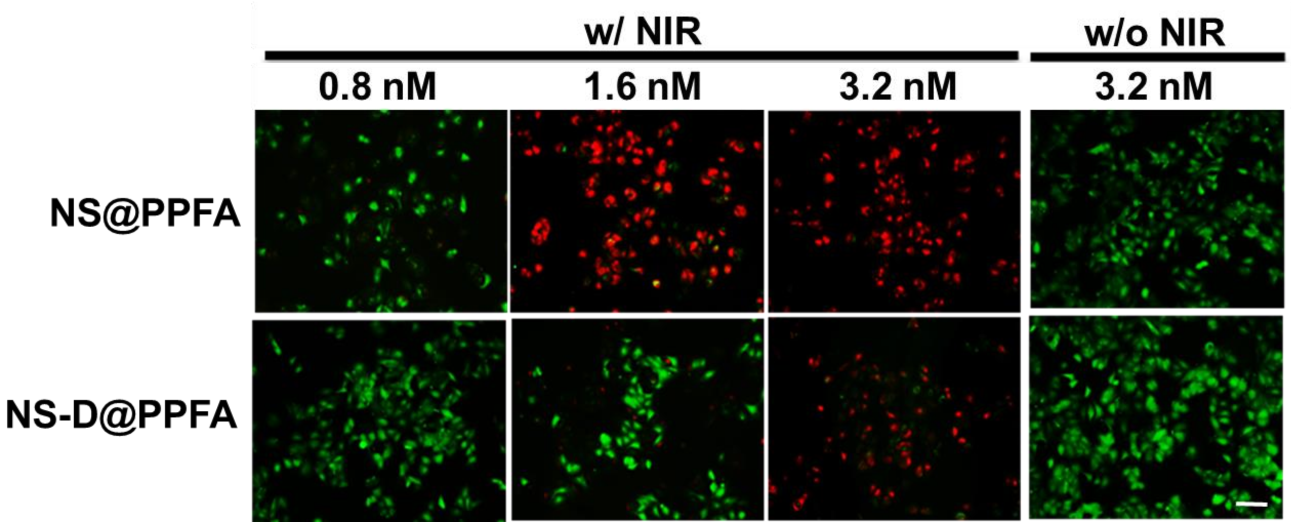

Figure S11. The live and dead assay of (A) NS@PPFA and (B) NS-D@PPFA in MCF-7 cells. Cells were incubated with varying concentrations (0.8 – 3.2 nM) of nanoagents for 6 h. After washing, laser irradiation was performed at energy density of (A) 0.9 W/cm^2 for 10 min and (B) 3.6 W/cm^2 for 3 min, respectively. Calcein-AM stains live cells green, while PI stains dead cells red. Scale bar: 50 μm.

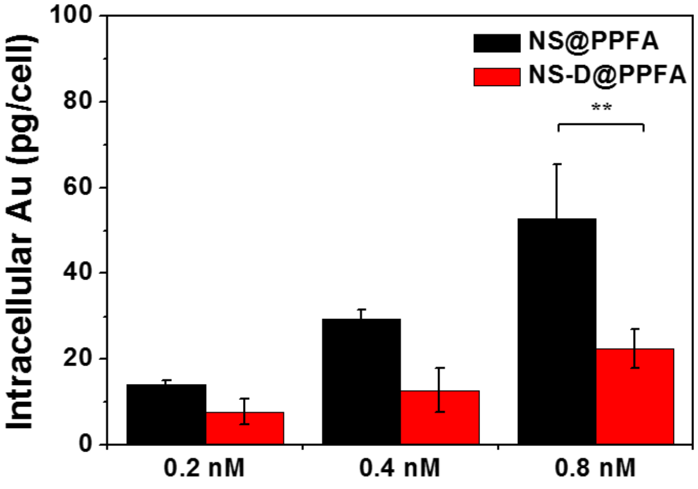

Figure S12. The quantitative analysis of intracellular Au in MCF-7 cells determined by ICP-MS. Cells were incubated with different nanoagents of serial concentrations (0.2 – 0.8 nM) for 2 h. $^{**}p < 0.01$.

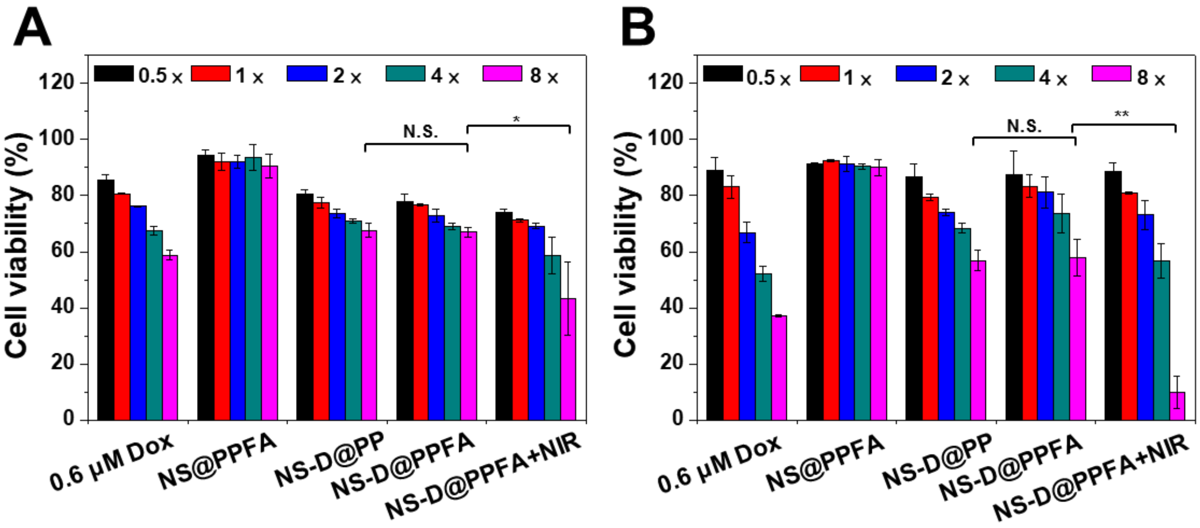

Figure S13. Cytotoxicity assay in (A) NIH/3T3 and (B) HaCaT cells. Cells were incubated with different therapeutic nanoagents (1× = 0.4 nM NS) for 12 h. After washing, cells were irradiated with NIR laser (3.6 W/cm^2) for 10 min and recovered in fresh medium for additional 24 h. *$p < 0.05$, **$p < 0.01$.

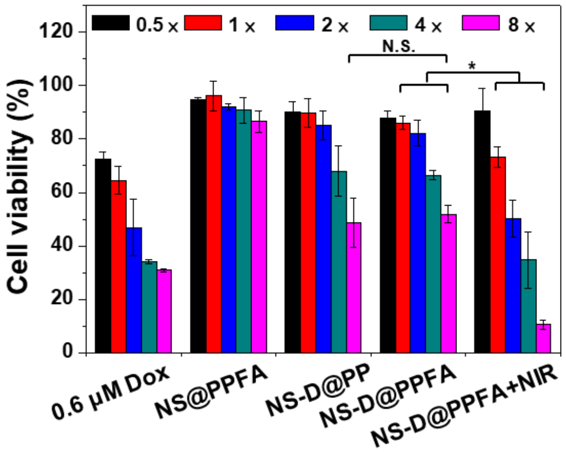

Figure S14. Cytotoxicity assay in HUVECs. Cells were incubated with different therapeutic nanoagents (1× = 0.4 nM NS) for 12 h. After washing, cells were irradiated with NIR laser (3.6 W/cm^2) for 10 min and recovered in fresh medium for additional 24 h. *$p < 0.05$.

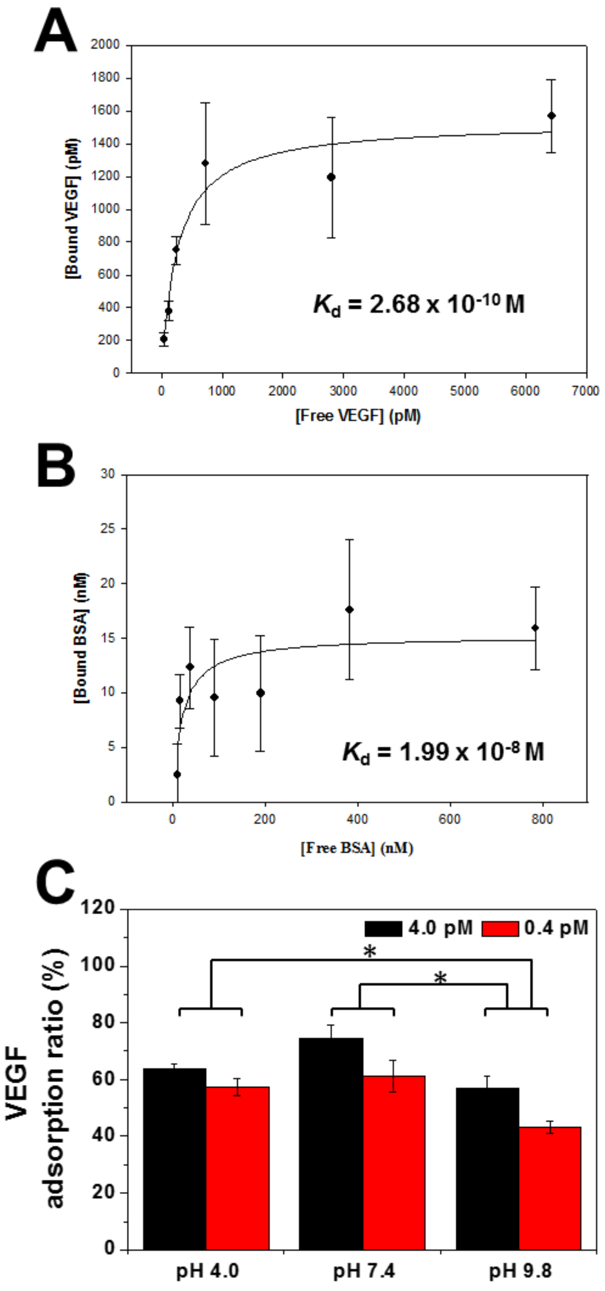

Figure S15. Binding affinity of NS@PPFA toward VEGF. The adsorption isotherms of NS-D@PPFA incubated with serial concentrations of (A) VEGF and (B) BSA in PBS (pH 7.4) contenting 10% FBS for 12 h. BSA was used as a control. K_d was determined using Sigma Plot software. (C) The VEGF adsorption of NS@PPFA in various pH solution. NS@PPFA was incubated with VEGF (1 nM) in PBS (10% FBS) of pH 4.0, 7.4, and 9.8 for 12 h, respectively.

VEGF ELISA Kit and fluorescence measurement was used to quantify the adsorbed amount of VEGF and FITC-BSA, respectively. *$p < 0.5$.

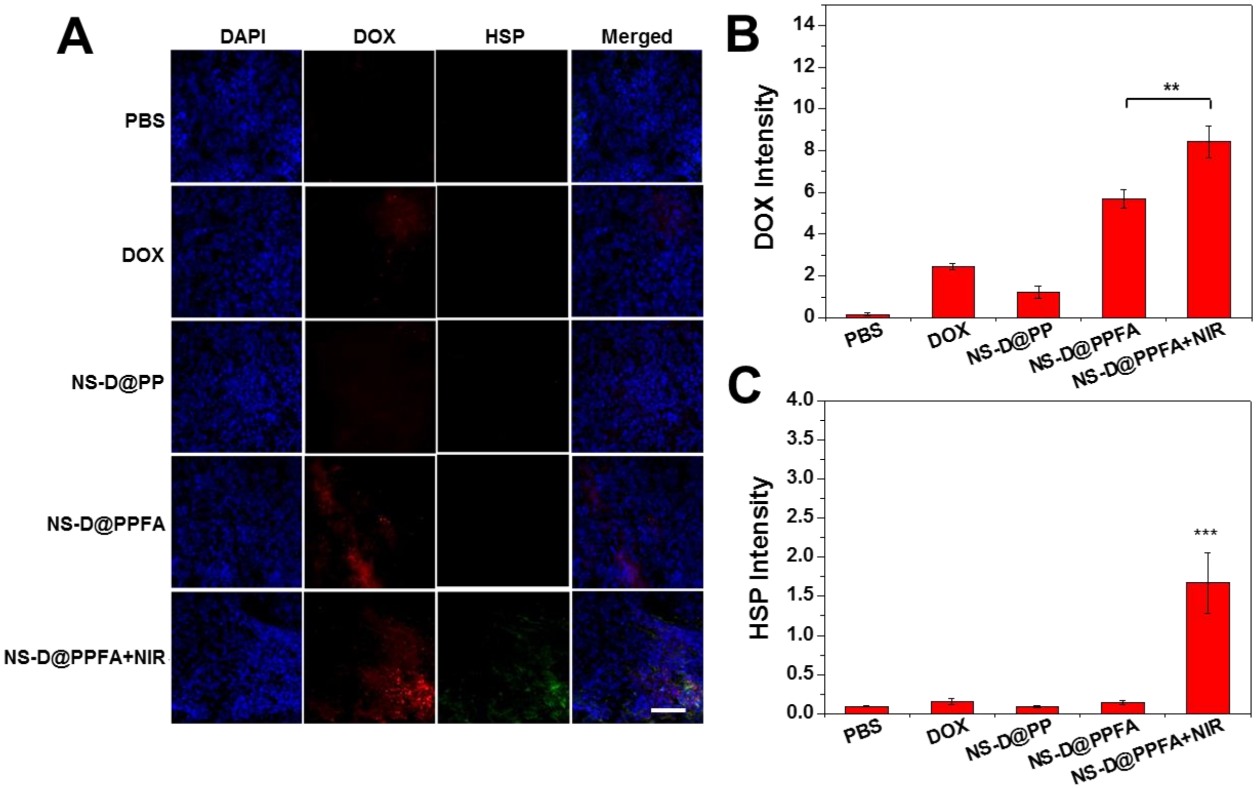

Figure S16. (A) Fluorescence (DOX and HSP70) staining microscopic images of tumor tissue sections harvested from MCF-7/ADR tumor-bearing nude mice injected intravenously with PBS, DOX (5 mg/kg), NS-D@PP, NS-D@PPFA and NS-D@PPFA with NIR irradiation (6 mg/kg Au, 1.8 mg/kg DOX), respectively. Laser treatment (808 nm, 0.9 W/cm^2, 3 min, 3 times) was performed twice at 24 and 48 h post-injection. Images of the tumor sections were acquired at 4 h after the 2nd irradiation (scale bar: 50 μm). Quantification of the fluorescence intensity of (B) DOX and (C) HSP70 in microscopic images of resistant breast cancer tissue using ImageJ software. Mean fluorescence within a ROI was measured and normalized by DAPI fluorescence intensity. Data represented as mean ± SD, n=3. $p^{**} < 0.01$, $p^{***} < 0.001$.

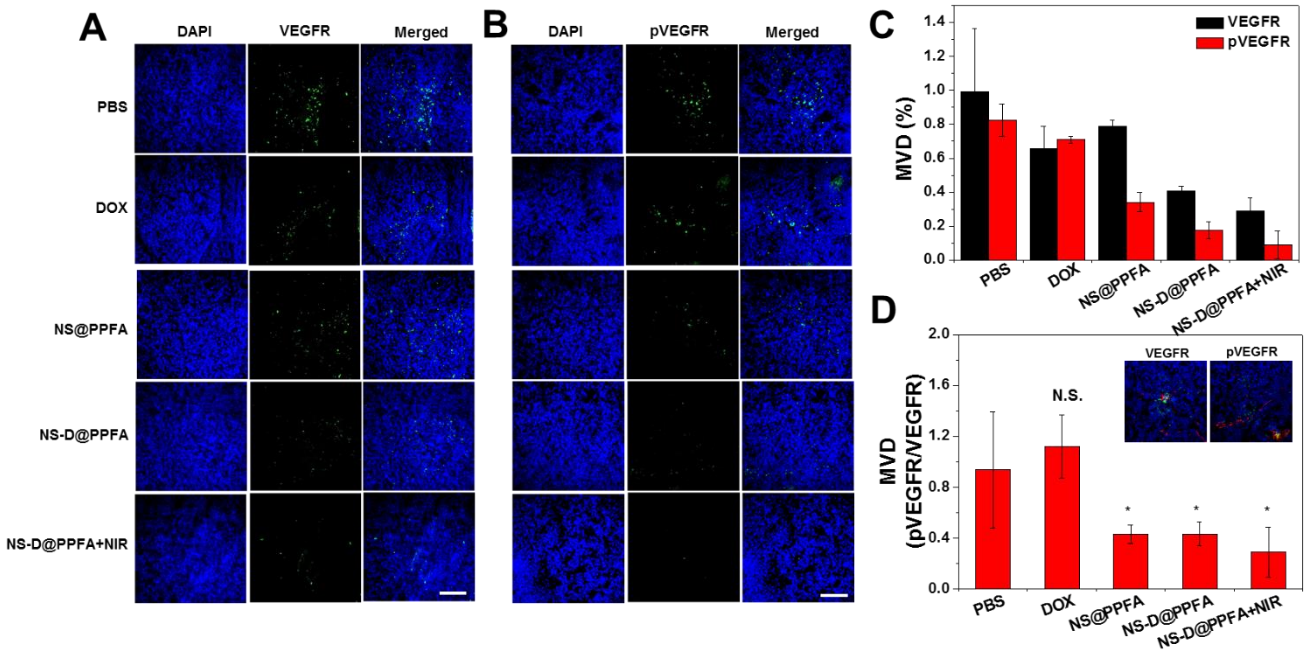

Figure S17. The immunofluorescence of (A) VEGFR2 and (B) pVEGFR2 staining of tumors after administration with PBS, DOX (5 mg/kg), NS@PPFA, NS-D@PPFA and NS-D@PPFA with NIR irradiation (6 mg/kg Au, 1.8 mg/kg DOX), respectively. Laser treatment (808 nm, 0.9 W/cm^2, 3 min, 3 times) was performed twice at 24 and 48 h post-injection. Images of the tumor sections were acquired at 4 h after the 2nd irradiation (scale bar: 50 μm). Evaluating the (C) microvessel density (MVD, %) and (D) ratio of pVEGFR to VEGFR of resistant breast tumor xenografts after different treatments. $p^* < 0.05$ versus PBS control. Inset shows the neovasculatures in the tumor tissue sections stained by CD31 (green) and VEGFR and pVEGFR (red), respectively. Scale bar = 50 μm.
